# Transcription Repressor Protein ZBTB25 Associates with HDAC1-Sin3a Complex in Mycobacterium tuberculosis-Infected Macrophages, and Its Inhibition Clears Pathogen by Autophagy

**DOI:** 10.1128/mSphere.00036-21

**Published:** 2021-02-24

**Authors:** Aravind Madhavan, K. B. Arun, Akhil Raj Pushparajan, M. Balaji, Ramakrishnan Ajay Kumar

**Affiliations:** a Mycobacterium Research Laboratory, Rajiv Gandhi Centre for Biotechnology, Thiruvananthapuram, Kerala, India; Washington University School of Medicine in St. Louis

**Keywords:** epigenetic modifications, ZBTB25, HDAC1, autophagy, interleukin-12, histone deacetylase, host-pathogen interactions

## Abstract

Downregulation of host gene expression is a key strategy employed by intracellular pathogens for their survival in macrophages and subsequent pathogenesis. In a previous study, we have shown that histone deacetylase 1 (HDAC1) levels go up in macrophages infected with Mycobacterium tuberculosis, and it hypoacetylates histone H3 at the promoter of *IL-12B* gene, leading to its downregulation. We now show that after infection with M. tuberculosis, HDAC1 is phosphorylated, and the levels of phosphorylated HDAC1 (pHDAC1) increase significantly in macrophages. We found that transcriptional repressor protein zinc finger and BTB domain 25 (ZBTB25) and transcriptional corepressor Sin3a associate with the HDAC1 silencing complex, which is recruited to the promoter of *IL-12B* to downregulate its expression in infected macrophages. Knocking down of *ZBTB25* enhanced release of IL-12p40 from infected macrophages. Inhibition of HDAC1 and ZBTB25 promoted colocalization of M. tuberculosis and LC3 (microtubule-associated protein 1A/1B-light chain 3) in autophagosomes. Induction of autophagy resulted in the killing of intracellular M. tuberculosis. Enhanced phosphorylation of JAK2 and STAT4 was observed in macrophages upon treatment with HDAC1 and ZBTB inhibitors, and inhibition of JAK2/STAT4 negated the killing of the intracellular pathogen, suggesting their role in the autophagy-mediated killing of intracellular M. tuberculosis. In view of the emergence of drug resistance in M. tuberculosis, host-directed therapy is an attractive alternative strategy to combat tuberculosis (TB). HDACs have been proposed to be host targets for TB treatment. Our study indicates that ZBTB25, a functional subunit of the HDAC1/Sin3a repressor complex involved in *IL-12B* suppression, could be an alternative target for host-directed anti-TB therapy.

**IMPORTANCE** Following infection with M. tuberculosis, levels of HDAC1 go up in macrophages, and it is recruited to the promoter of *IL-12B* where it hypoacetylates histone H3, leading to the downregulation of the gene. Here, we show that host transcriptional repressor protein ZBTB25 and transcriptional corepressor Sin3a associate with HDAC1 in the silencing complex. Knocking down of *ZBTB25* prevented the recruitment of the complex to the promoter and consequently enhanced the gene expression and the release of IL-12p40 from infected macrophages. Pharmacological inhibition of ZBTB25 in infected macrophages resulted in the induction of autophagy and killing of intracellular M. tuberculosis. Drug-resistant TB is a serious challenge to TB control programs all over the world which calls for finding alternative therapeutic methods. Host-directed therapy is gaining significant momentum in treating infectious diseases. We propose that ZBTB25 is a potential target for host-directed treatment of TB.

## INTRODUCTION

Tuberculosis (TB) caused by Mycobacterium tuberculosis is a prominent cause of death worldwide, especially in tropical regions. Although effective anti-TB drugs and the BCG vaccine have been in use for almost a century, TB still remains a global malady that cannot be eradicated. The current therapeutic drugs inhibit M. tuberculosis by interfering with critical pathways such as DNA metabolism and mycolic acid biosynthesis. Typically, treatment of TB involves administration of multiple drugs over a period of 4 to 6 months. This, combined with the side effects of the drugs, leads to noncompliance, which is one major reason for emergence of drug-resistant TB. The ability of M. tuberculosis to remain within the host in a dormant state for a long time, and to get reactivated when the body’s defenses are compromised, is another reason for the success of M. tuberculosis as a recalcitrant pathogen. Novel and fast-acting anti-TB molecules against metabolically active and dormant bacteria are required for effective control of TB. In recent times, however, an alternative approach, namely, host-directed therapy, has emerged as a promising treatment method which, unlike the current anti-TB drugs, targets host factors which facilitate the survival and pathogenicity of intracellular M. tuberculosis (1, 2).

After invading the host, M. tuberculosis inhabits membrane-bound phagosomes and employs mechanisms to evade the innate immune responses that trigger macrophages (3, 4). After phagocytosis, the primary host defense mechanism is initiated by the secretion of proinflammatory cytokines tumor necrosis factor alpha (TNF-α), interleukin-1 beta (IL-1β), IL-6, and IL-12. Proinflammatory cytokines play a key role in initiating antimicrobial responses (5–7). The T-cell-mediated immunity augments the capability of macrophages to clear bacteria by activating Th1 responses and boosting their cytotoxic activity (8).

Recent studies reveal that intracellular pathogens suppress host immune functions by targeting the host chromatin and epigenetic regulators (9). In a previous study, we had shown that infection of human macrophages with virulent M. tuberculosis causes the levels of histone deacetylase 1 (HDAC1) to increase significantly and repress expression of the *IL-12B* gene (10). HDAC1 has been shown by numerous studies to be the catalytic core of many corepressor complexes involved in the downregulation of eukaryotic gene expression.

Microbial pathogens have developed several strategies to take control of the host immune system. Suppression of host gene expression by HDACs plays a significant role in keeping the host immune system in check. Epstein-Barr virus proteins TRF2 (11) and EBNA3C (12) bind to the HDAC1 complex, and these interactions are necessary for viral replication in the host. Terhune et al. (13) identified that cellular proteins RBBP4 and CHD4 and human cytomegalovirus (HCMV) protein pUL29/28/38 interact with HDAC1 upon HCMV infection to stimulate the accumulation of immediate early viral RNAs. 2-aminoacetophenone, a quorum-sensing molecule from Pseudomonas aeruginosa, regulates host HDAC1 expression and enhances the interaction between cellular p50 protein and HDAC1, leading to repression of proinflammatory responses (14). The ankyrin molecule from Anaplasma phagocytophilum induces the recruitment of the HDAC1 complex to promoters of antimicrobial defense genes (15). Our own study has shown that HDAC1 is recruited to the promoter of *IL-12B* to downregulate its expression in M. tuberculosis-infected macrophages (10). However, it remains to be seen if M. tuberculosis proteins are associated with the complex in infected cells. Also, the identity of host proteins associated with HDAC1 during M. tuberculosis infection and their role in the progress of infection/pathogenesis are also not known. Although our study has shown that knocking down or inhibiting HDAC1 resulted in the enhanced killing of M. tuberculosis, we propose that inhibition of HDAC1-associated proteins, rather than inhibiting HDAC1 itself, which has a broader role in cellular gene regulation, may be a more specific strategy for host-directed anti-TB therapy.

The present study, in which we analyzed the HDAC1-associated proteins in M. tuberculosis-infected macrophage cells, reveals that ZBTB25, a repressive transcription factor, associates with HDAC1 repressor complex. ZBTB25 is a member of the broad complex, tram track, bric-a-brac/poxvirus and zinc finger (BTB/POZ) transcription family, and these proteins possess a DNA-binding zinc finger motif at the C terminus and a protein-binding BTB/POZ domain at the N terminus. The zinc finger identifies and binds to the specific DNA sequences, while the BTB/POZ domain helps in the homodimerization and/or heterodimerization and interaction with other proteins (16). The human genome encodes about 60 genes under the ZBTB family (17), although the function of most of the members is yet to be ascertained. In the present study, we show that ZBTB25, along with HDAC1, binds to the *IL-12B* gene promoter and represses its expression. Inhibition of ZBTB25 derepresses the expression of *IL-12B* gene and also activates autophagy-associated genes. Because of its dual role in HDAC1-mediated repression of genes and autophagy, we propose that ZBTB25 may be a potential therapeutic target for TB treatment.

## RESULTS

### HDAC1 in macrophages is phosphorylated upon M. tuberculosis infection.

**FIG 1:**
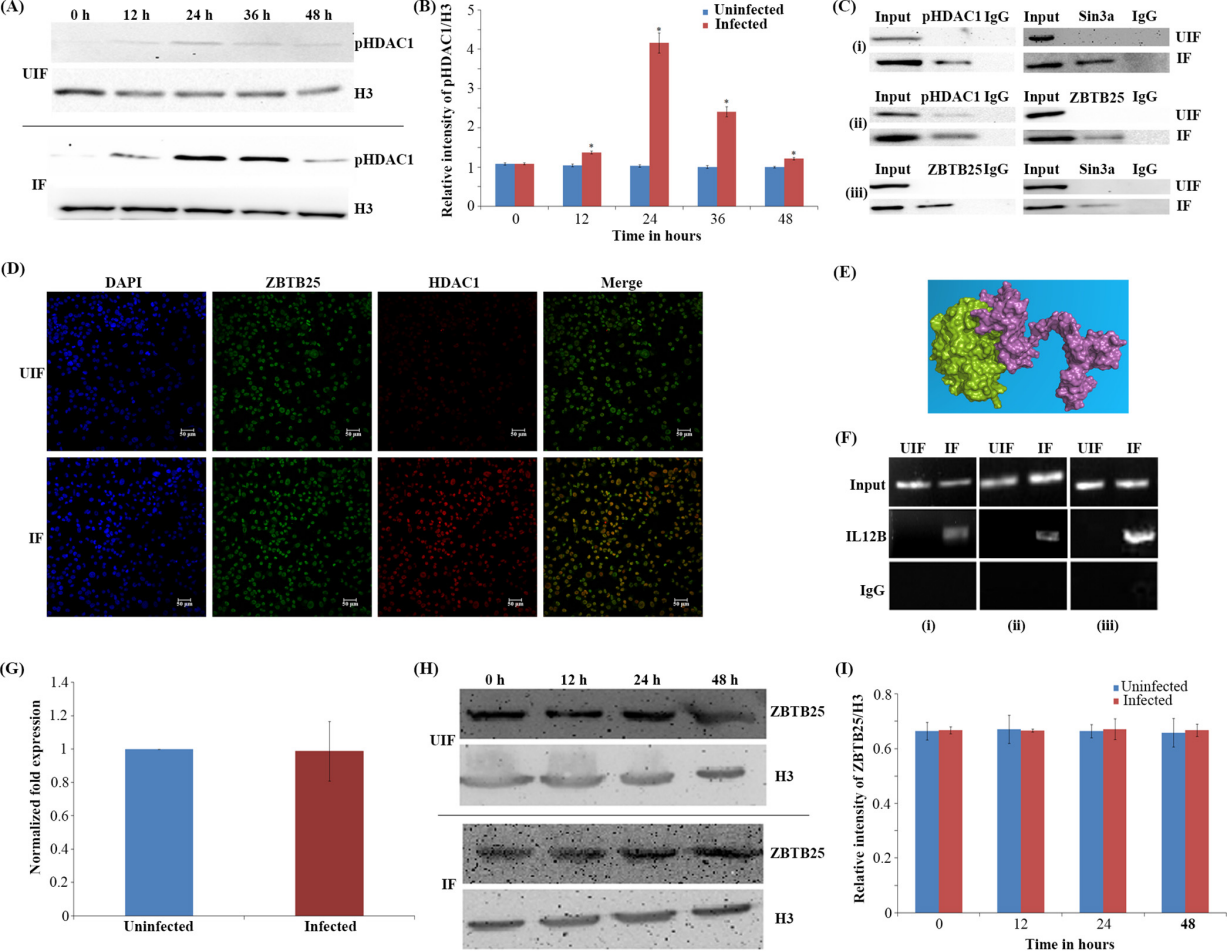
Phosphorylated HDAC1 interacts with ZBTB25, and they are recruited to the *IL-12B* promoter. (A) Time course of levels of phosphorylated HDAC1 in M. tuberculosis-infected macrophages. pHDAC1 levels in uninfected (UIF) and infected (IF) samples. (B) Densitometric analysis of pHDAC1 bands normalized with that of histone H3. Each value represents mean ± SD from triplicate measurements. *, significantly different from UIF sample; *P* ≤ 0.05. (C) Coimmunoprecipitation analysis of the association between ZBTB25 and Sin3a-HDAC1 complex. Whole-cell lysates were immunoprecipitated with the antibodies against (i) ZBTB25, (ii) Sin3a, and (iii) HDAC1. IgG was used as the negative control. Immunocomplexes were then probed with antibodies as indicated. (D) Immuno-cytochemical imaging shows HDAC1 colocalizes with ZBTB25 inside the nucleus of macrophages infected with M. tuberculosis. The cells were visualized by confocal microscopy at 24 hpi. (E) Docking analysis shows that HDAC1 can interact with ZBTB25. (F) Status of ZBTB25, HDAC1, and Sin3a recruitment on the *IL-12B* promoter by ChIP. ChIP with (i) ZBTB25, (ii) HDAC1, and (iii) Sin3a antibodies. (G) Status of expression of *ZBTB25* in macrophages upon M. tuberculosis infection by real-time PCR; (H) protein levels by Western blotting. (I) Densitometric analysis of ZBTB25 bands normalized with that of histone H3. Each value represents mean ± SD from triplicate measurements.

To delineate if HDAC1 is phosphorylated during M. tuberculosis infection, human macrophages were infected with M. tuberculosis H37Rv, and a time course analysis of phosphorylated HDAC1 (pHDAC1) and nonphosphorylated HDAC1 was carried out from 0 to 48 h by Western blot analysis of uninfected and M. tuberculosis-infected macrophages. At 24 h postinfection (hpi), the levels of pHDAC1 increased by 4-fold in macrophages infected with M. tuberculosis (Fig. 1A and B) compared to the uninfected cells. Time course of the levels of nonphosphorylated HDAC1 in infected and uninfected cells was analyzed by Western blotting (Fig. S1A and B at https://rgcb.res.in/documents/publication/drajay/mSPhere00036-21%20supplemental%20file%20RGCB.pdf).

To find out whether it is the pHDAC1 that is recruited to the *IL-12B* promoter in macrophages during M. tuberculosis infection, we performed a chromatin immunoprecipitation with antibodies against pHDAC1 followed by PCR for the promoter sequence. Amplification of the *IL-12B* promoter region confirmed the recruitment of pHDAC1 to it (Fig. 1F, panel ii).

### Phosphorylated HDAC1 interacts with ZBTB25, and both are recruited to the *IL-12B* promoter.

To identify the interacting partners of HDAC1 during M. tuberculosis infection, we infected THP-1-derived macrophages with M. tuberculosis H37Rv and immunoprecipitated pHDAC1-associated proteins from cell extracts with pHDAC1-specific (serine 421 or serine 423) polyclonal antibodies. Subsequent liquid chromatography-tandem mass spectrometry (LC-MS/MS) analysis detected and identified HDAC1 and two other macrophage proteins, namely, ZBTB25 and Sin3a (Table S1 at https://rgcb.res.in/documents/publication/drajay/mSPhere00036-21%20supplemental%20file%20RGCB.pdf) in the precipitate. Interaction of ZBTB25 with HDAC1 and Sin3a was confirmed by reciprocal coimmunoprecipitations (Fig. 1C). To further investigate the association of HDAC1 with ZBTB25, we carried out an immunofluorescence microscopy analysis. Confocal images of the macrophages infected with M. tuberculosis showed that ZBTB25 and HDAC1 colocalize within the nucleus at 24 hpi (Fig. 1D), which was not observed in uninfected cells. Interaction of the HDAC1 and ZBTB25 was analyzed employing *in silico* docking analysis, which showed that HDAC1 and ZBTBT25 interact with each other strongly, with a docking score of −914.5 Kcal/mol (Fig. 1E; Fig. S1C and D and Table S3 at https://rgcb.res.in/documents/publication/drajay/mSPhere00036-21%20supplemental%20file%20RGCB.pdf). Following infection, ZBTB25 and Sin3a were found to be recruited to the *IL-12B* promoter at 24 hpi. This was confirmed by chromatin immunoprecipitation of macrophage DNA with antibodies against ZBTB25 and Sin3a, followed by a PCR for these promoter sequences (Fig. 1F). The levels of ZBTB25 in the uninfected and infected macrophages were evaluated using real-time PCR, and a time course analysis was done using Western blotting with ZBTB25-specific antibodies (Fig. 1G). We found no significant difference in the levels of ZBTB25 in M. tuberculosis-infected and uninfected macrophages (Fig. 1H and I).

### Knocking down of ZBTB25 restores the expression of *IL-12B* and reduces the intracellular survival of M. tuberculosis.

**FIG 2:**
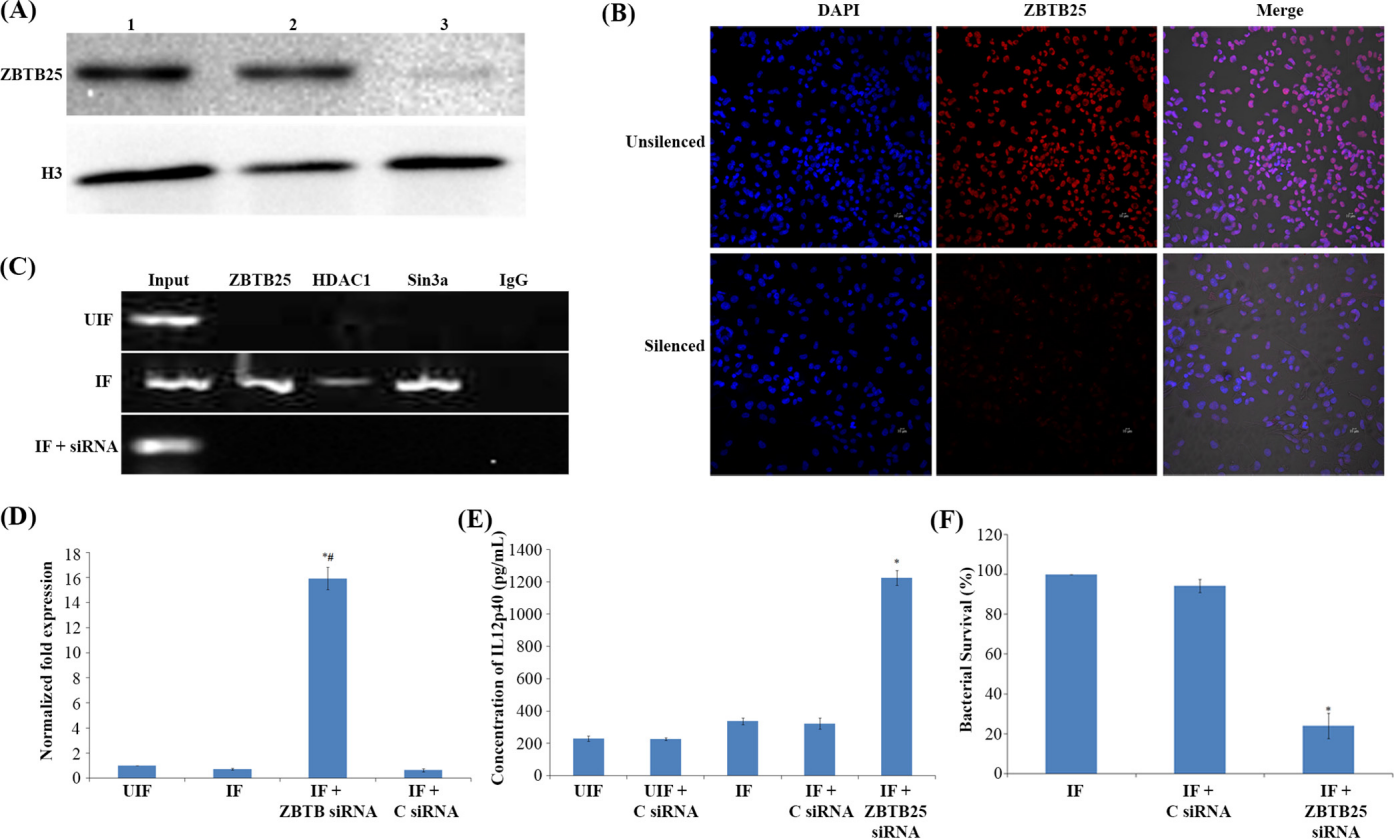
Knocking down of ZBTB25 enhances IL-12p40 and reduces the intracellular survival of M. tuberculosis. THP-1 macrophage cells were transfected with *ZBTB25* siRNA, or scrambled siRNA control using HiPerFect transfection reagent. (A) Western blot analysis of macrophage cells in which the expression of endogenous *ZBTB25* was knocked down using *ZBTB25* siRNA. Lane 1, normal macrophages; lane 2, macrophages transfected with scrambled siRNA control; lane 3, macrophages transfected with *ZBTB25* siRNA. (B) Immuno-cytochemical staining with anti-ZBTB25 antibody and DAPI of control THP-1 macrophages. Cells in which *ZBTB25* was knocked down showed reduced fluorescence compared to the control macrophages. (C) Knocking down of *ZBTB25* blocks the recruitment of the HDAC1 silencing complex to the *IL-12B* promoter. Status of (i) ZBTB25, (ii) HDAC1, and (iii) Sin3a on the *IL-12B* promoter by ChIP followed by PCR. UIF, normal macrophages; IF, macrophages transfected with scrambled siRNA and infected with M. tuberculosis; IF+siRNA, macrophages transfected with *ZBTB25* siRNA and infected with M. tuberculosis. (D) *IL-12B* expression is upregulated in M. tuberculosis-infected macrophages upon ZBTB25 inhibition. qPCR of *IL-12B* when *ZBTB25* is knocked down. *#, *IL-12B* expression is significantly different from uninfected (UIF) and infected (IF) samples; *P* ≤ 0.05. (E) ELISA of IL-12p40 when *ZBTB25* is knocked down in macrophages. *, IL-12p40 levels are significantly different from infected (IF) sample; *P* ≤ 0.05. (F) Survival of M. tuberculosis decreases in macrophages when *ZBTB25* is knocked down. Macrophages in which *ZBTB25* was knocked down were infected with M. tuberculosis. Macrophages not treated with siRNA but infected with M. tuberculosis were also kept as control. Intracellular bacterial viability was determined by counting the number of CFU. Data shown here are the mean ± SD of three independent experiments. *, survival is significantly different from both controls; *P* ≤ 0.05.

Since we observed an interaction between HDAC1 and ZBTB25, we tested whether ZBTB25 plays a role in the survival of M. tuberculosis inside the macrophages. To investigate this, we knocked down *ZBTB25* in THP-1-derived macrophages and infected them with virulent M. tuberculosis. The efficiency of knockdown was confirmed by quantitative reverse transcription-PCR (qRT-PCR), Western blotting, and confocal microscopy (Fig. 2A and B and Fig. S1E). We observed that ZBTB25, HDAC1, and Sin3a were not recruited to the promoter of *IL-12B* in macrophages in which ZBTB25 was knocked down (Fig. 2C). As expected, knockdown of *ZBTB25* significantly enhanced *IL-12B* mRNA and IL-12p40 protein levels in infected cells as confirmed by qRT-PCR (Fig. 2D) and enzyme-linked immunosorbent assay (ELISA) (Fig. 2E). Colony forming units (CFU) of intracellular M. tuberculosis were enumerated by lysing the infected macrophages at 24 hpi and plating them on Middlebrook 7H10 agar. Macrophages treated with scrambled small interfering RNA (siRNA) and infected with M. tuberculosis served as control. A significant decrease was observed in the number of CFU in macrophages in which *ZBTB25* was knocked down (Fig. 2F).

### Treatment of infected macrophages with DP and CI994 enhances intracellular clearance of M. tuberculosis.

The zinc finger domain of ZBTB25 is important for its ability to bind DNA. Different zinc ejector drugs, dithiopyridine (DP), disulfiram (DS), and 2-nitrobenzoic acid (2NB), were tested for their ability to inhibit and eject the zinc ion from the recombinant ZBTB25 protein. The most effective Zn ejector was selected based on its efficiency to eject zinc from the protein (Fig. S2A at https://rgcb.res.in/documents/publication/drajay/mSPhere00036-21%20supplemental%20file%20RGCB.pdf). The Zn^2+^ ions released by the Zn ejectors were monitored using the fluorescence of the zinc-specific fluorophore FluoZin-3. For a 50% Zn^2+^ ejection from ZBTB25, the concentrations required for DP, DS, and 2NB were 4.61 ± 0.28 μM, 10.35 ± 0.30 μM, and 25.54 ± 0.37 μM, respectively, as determined by time course experiments with different concentrations of the drugs (Fig. S2A and B). These results suggested that DP is a strong zinc ejector and inhibits ZBTB25 significantly compared to other Zn ejector molecules used in this study. The 3-(4,5-dimethylthiazole-2-yl)-2,5-diphenyl tetrazolium bromide (MTT) cytotoxicity analysis showed that DP is less toxic to THP-1 cells (Fig. S2C). Even after exposure to 30 μM DP for 24 h, 85.33 ± 1.53% of the cells remained viable.

To find the concentration of DP for the inhibition of ZBTB25 protein in M. tuberculosis*-*infected macrophages, we exposed the infected THP-1 cells to different concentrations of the same (5, 10, 15, and 20 μM) and monitored the recruitment of ZBTB25 to the *IL-12B* promoter. Recruitment of ZBTB25 was completely abolished at 20 μM concentration of DP (Fig. S2D and E). We also tested the intracellular survival of M. tuberculosis after treatment of infected THP-1 cells with different concentrations of DP (5, 10, 15, 20, and 30 μM). The intracellular survival (Fig. S2F) suggested that viability of intracellular bacteria was reduced significantly after treatment of infected THP-1 cells with 20 and 30 μM DP (35.11 ± 4.32% and 31.71 ± 4.79%, respectively) at 24 hpi. Based on these results, 20 μM DP was used for further assays in this study.

In addition, we evaluated the effect of CI994, an HDAC1 inhibitor, on intracellular survival of M. tuberculosis. Specificity of CI994 toward HDAC1, HDAC2, and HDAC3 was tested using HDAC inhibitor screening assay kit (Cayman Chemical, USA) based on the manufacturer’s protocol (Fig. S3A at https://rgcb.res.in/documents/publication/drajay/mSPhere00036-21%20supplemental%20file%20RGCB.pdf). The 50% inhibitory concentration (IC_50_) values of CI994 for HDAC1, HDAC2, and HDAC3 were found to be 0.52 ± 0.03 μM, 1.09 ± 0.07 μM, and 1.54 ± 0.09 μM, respectively. The MTT cytotoxicity analysis showed that CI994 is not toxic to THP-1 cells (Fig. S3B). The recruitment of HDAC1 to the *IL-12B* promoter in infected macrophages was monitored at concentrations of 0.5, 10, 15, and 20 μM, and it was observed that HDAC1 recruitment was decreased by 2.6-fold at 15 and 20 μM concentrations of CI994 compared to the lower concentrations used in the experiment (Fig. S3C and D). Intracellular survival of M. tuberculosis was tested at different concentrations of CI994 (0.5, 5, 10, 15, and 20 μM). The results showed that CI994 at 15 μM is effective in reducing the intracellular survival of M. tuberculosis by 47.39 ± 2.42% at 24 hpi (Fig. S3E), and therefore, this concentration was used in subsequent experiments.

**FIG 3:**
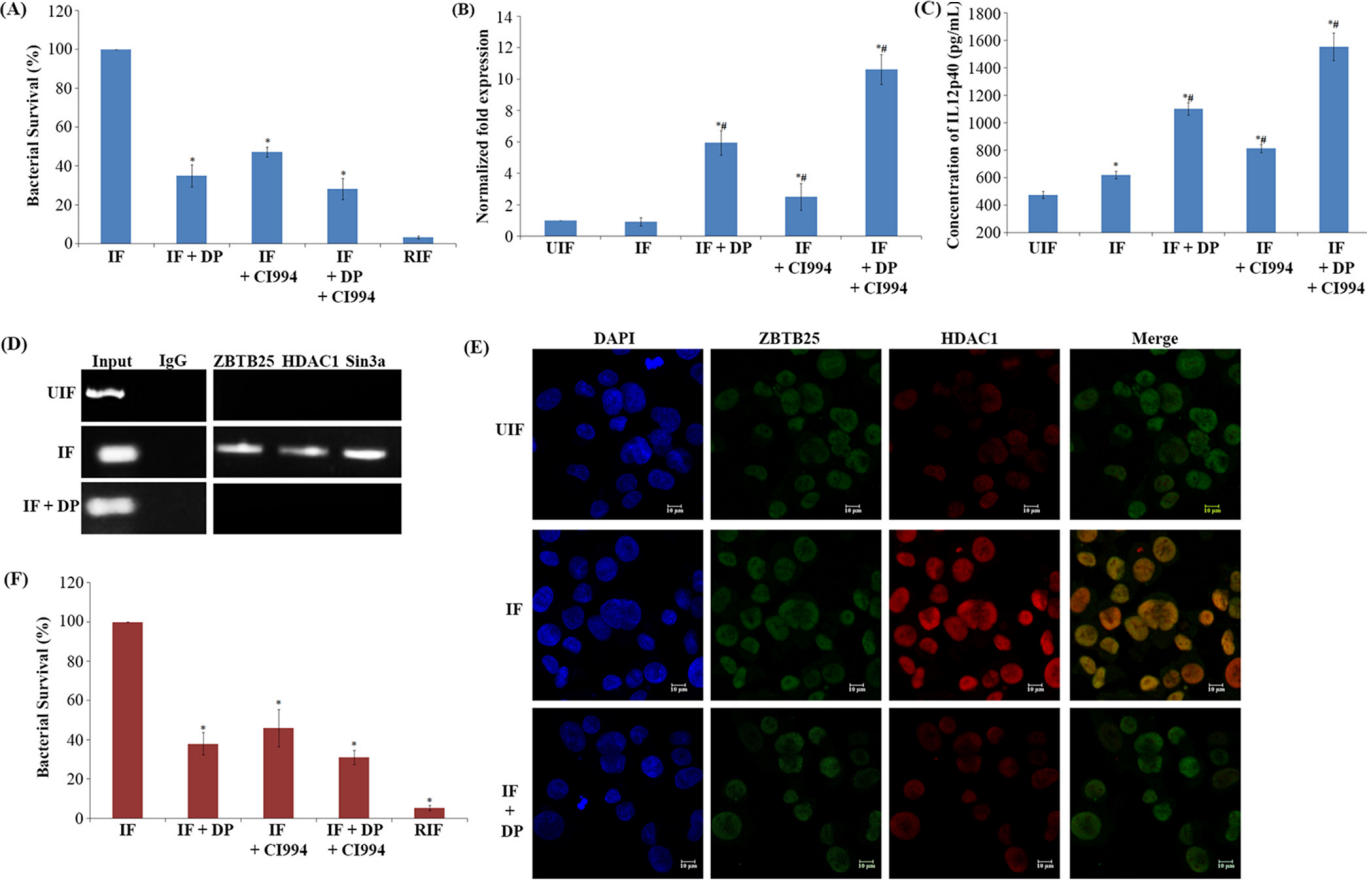
Treatment of infected macrophages with DP (ZBTB25 inhibitor) and CI994 (HDAC1 inhibitor) enhanced the intracellular clearance of M. tuberculosis. THP-1-derived macrophages were infected with M. tuberculosis H37Rv. After M. tuberculosis infection, cells were treated with DP (20 μM) and/or CI994 (15 μM) or rifampicin (1 μg/ml) for 24 h. (A) Intracellular bacterial viability was determined based on the number of CFU. Isolation of bacilli from the macrophages was carried out at 24 h. The number of viable bacilli in each of the plate was assayed by plating lysed macrophages on 7H10 agar plates and incubating the plates at 37°C for 3 weeks and counting the CFU. Values are shown from 3 independent experiments (mean ± SD). *, survival is significantly different from infected samples (IF); *P* ≤ 0.05. (B) qPCR for the expression of the *IL-12B* mRNA transcript (normalized to β-actin mRNA expression). *#, *IL-12B* expression is significantly different from uninfected (UIF) and infected samples (IF), respectively; *P* ≤ 0.05. (C) ELISA of IL-12p40 after inhibitor treatment. *#, IL-12p40 levels are significantly different from uninfected (UIF) and infected samples (IF), respectively; *P* ≤ 0.05. (D) DP treatment blocks the recruitment of the HDAC1 silencing complex to the *IL-12B* promoter. Status of (i) ZBTB25, (ii) HDAC1, and (iii) Sin3a on the *IL-12B* promoter by ChIP PCR. (E) Immuno-cytochemical imaging shows HDAC1 does not colocalize with ZBTB25 inside the macrophage nucleus after DP treatment. (F) Intracellular viability of M. tuberculosis in infected PBMC after treatment with DP, CI994, and rifampicin was determined by counting the number of CFU. *, survival is significantly different from infected sample (IF); *P* ≤ 0.05.

Interestingly, treatment with a combination of DP and CI994 showed an enhanced effect on the expression of *IL-12B*, IL-12p40 levels and the intracellular M. tuberculosis survival (Fig. 3A to C, respectively). The CFU count of combination treatment indicated a significant reduction in the number (72% killing) of M. tuberculosis inside the macrophages compared to the macrophages treated with CI994 alone (Fig. 3A). In the presence of DP, recruitment of HDAC1 complex proteins (HDAC1, ZBTB25, and Sin3a) to the *IL-12B* promoter was not observed (Fig. 3D).

Treatment with DP disrupted the interaction and colocalization of ZBTB25 and HDAC1 in the nucleus, which was confirmed by confocal microscopy (Fig. 3E). Toxicity of DP toward THP-1 cells was tested by MTT assay, and that toward M. tuberculosis H37Rv was tested by resazurin microtiter assay (REMA) (Fig. S3F and Fig. S4A to C at https://rgcb.res.in/documents/publication/drajay/mSPhere00036-21%20supplemental%20file%20RGCB.pdf). The drug concentrations used in this study were not toxic to THP-1-derived macrophages and M. tuberculosis at which it interfered with the recruitment of the repressor complex to the *IL-12B* promoter (Fig. S3F).

Consistent with our observation in THP-1-derived macrophages, we found a similar trend when peripheral blood mononuclear cells (PBMC) were infected with M. tuberculosis and treated with these inhibitors. Treatment with DP and CI994 resulted in the killing of 62% and 54% of M. tuberculosis, respectively (Fig. 3F), and the combination of DP and CI994 enhanced the killing of intracellular M. tuberculosis to 70% in PBMC. The levels of IL-12p40 were found to be significantly high in M. tuberculosis-infected macrophages treated with DP and CI994 compared to those in infected but untreated cells (Fig. S5 at https://rgcb.res.in/documents/publication/drajay/mSPhere00036-21%20supplemental%20file%20RGCB.pdf).

### Autophagy is activated when *IL-12B* expression is restored in macrophages.

**FIG 4:**
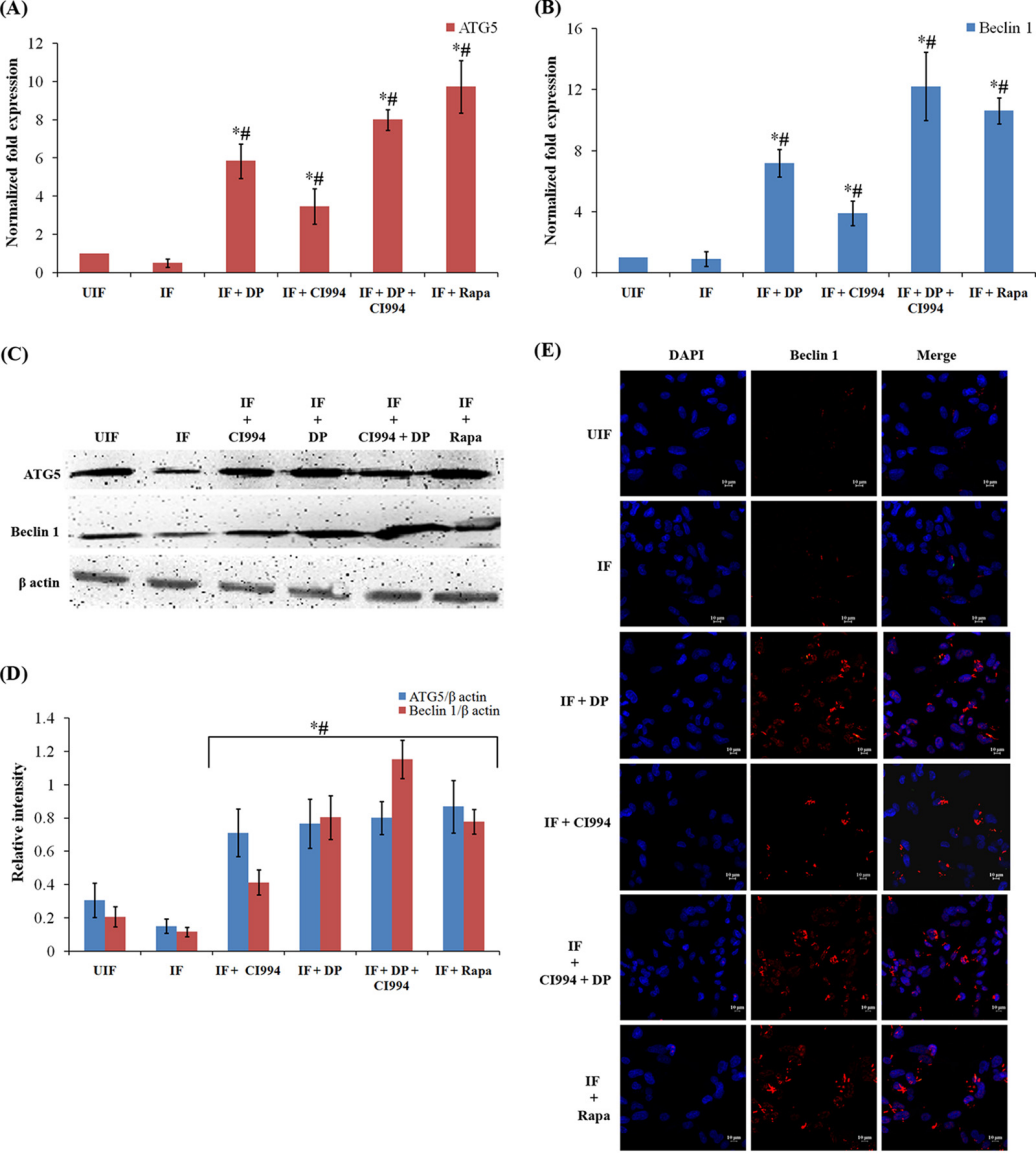
DP and CI994 induce autophagy in human macrophages. (A and B) DP and CI994 treatment of infected macrophages upregulated the expression of the autophagy-related genes *BECN1* and *ATG5*. Differentiated human THP-1 cells were infected with M. tuberculosis H37Rv for 4 h and treated with DP and CI994 or rapamycin (Rapa). The mRNA levels of autophagy-related genes *ATG5* (A) and *BECN1* (B) (normalized to β-actin expression) in THP-1-derived macrophages were measured by qPCR. Results are shown from 3 independent experiments (mean ± SD). *#, expression of *BECN1* and *ATG5* is significantly different from that in uninfected (UIF) and infected (IF) samples, respectively (*P* ≤ 0.05). (C) Western blotting of ATG5, Beclin 1, and β-actin. (D) Densitometric analysis of ATG5 and Beclin 1 bands normalized with that of β-actin. Each value represents mean ± SD from triplicate measurements. *#, band intensity of ATG5 and Beclin 1 is significantly different from that in uninfected (UIF) and infected (IF) samples, respectively (*P* ≤ 0.05). (E) Immuno-cytochemical imaging shows levels of Beclin 1 in variously infected macrophages at 24 hpi.

**FIG 5:**
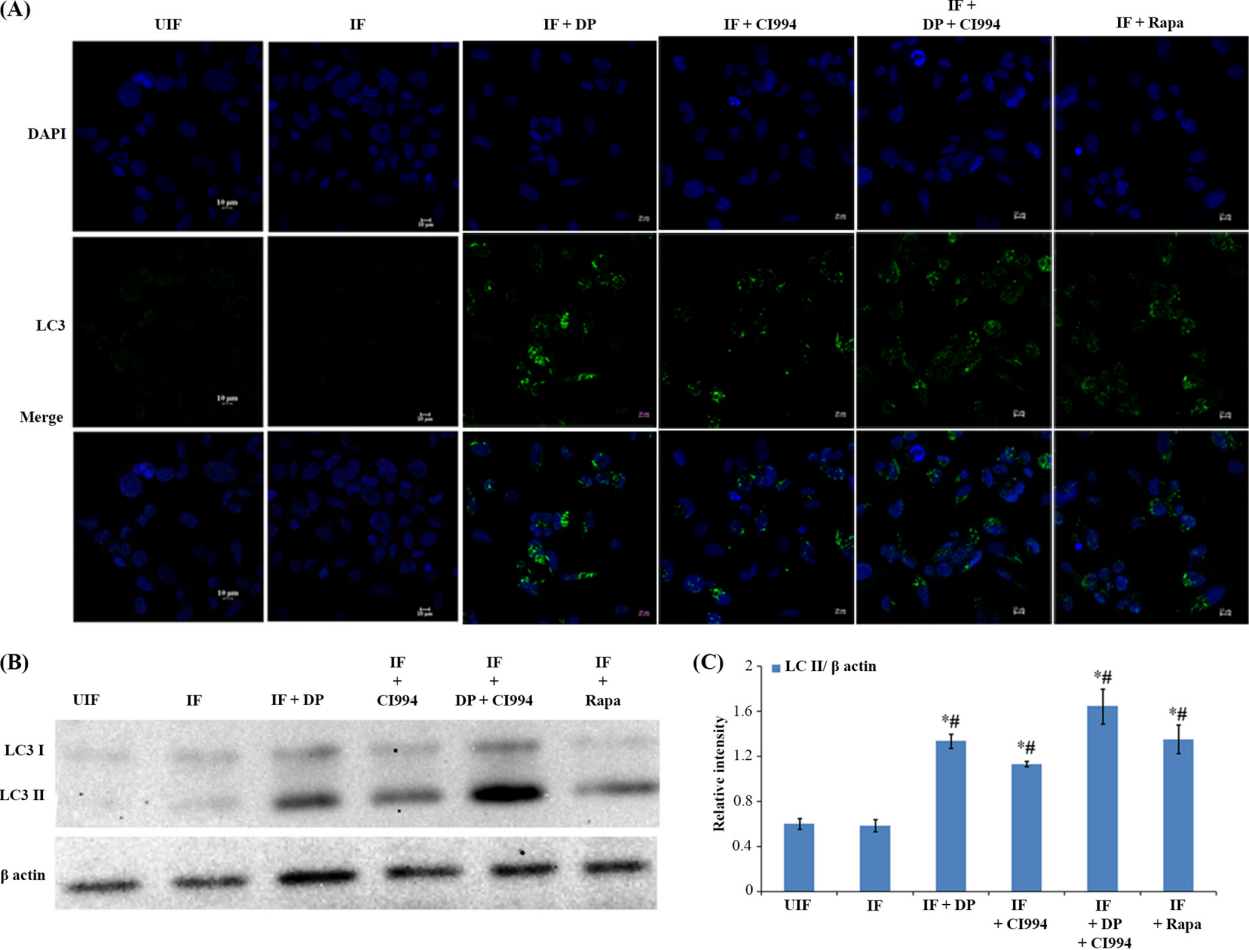
DP and CI994 induce autophagic clearance of bacteria by enhancing the expression of LC3 in human macrophages. THP-1-derived macrophages were infected with M. tuberculosis expressing GFP (for 4 h) and were treated with DP, CI994, and rapamycin (Rapa). (A) Cells were fixed and stained with DAPI to visualize the nuclei (blue) with anti-LC3 antibodies followed by the addition of FITC-conjugated mouse IgG (green). One representative immunofluorescence image out of 3 independent replicates is shown. (B) Representative Western blotting shows the conversion of LC3-I to LC3-II and β-actin from 3 independent experiments. (C) Densitometric analysis of LC3-II bands normalized with that of β-actin. Each value represents mean ± SD from triplicate measurements. *#, LC3 levels are significantly different from those in uninfected (UIF) and infected (IF) samples, respectively (*P* ≤ 0.05).

Treatment of macrophage with DP (20 μM) and CI994 (15 μM) significantly enhanced *IL-12B* expression and decreased the survival of M. tuberculosis. Since DP and CI994 are found to increase the IL-12p40 levels and since it is reported that IL-12 mediates killing of intracellular mycobacteria through autophagy, we investigated if DP and CI994 can induce autophagy in THP-1-derived macrophage cells. Autophagosome formation is regulated by two critical proteins, namely, ATG5 and Beclin 1. Expression of *BECN1* (encoding Beclin 1) and *ATG5* and their product levels were low (Fig. 4A and B) in macrophages infected with M. tuberculosis. However, when they were treated with DP alone or in combination with CI994, expression of both the genes and their protein levels were significantly elevated (Fig. 4A to D). Thus, treatment with DP and CI994 was able to overcome the M. tuberculosis-mediated suppression of the autophagy genes *ATG5* and *BECN1* in human macrophages. Confocal microscopy revealed that the levels of Beclin 1 increased after the drug treatment (Fig. 4E). Treatment of uninfected normal macrophages with DP and CI994 did not result in the elevation of the levels of Beclin 1 (Fig. S6A at https://rgcb.res.in/documents/publication/drajay/mSPhere00036-21%20supplemental%20file%20RGCB.pdf). Microtubule-associated protein 1A/1B-light chain 3 (LC3) is another key protein required for autophagosome formation and is considered an autophagy marker. M. tuberculosis-infected macrophages showed reduced levels of LC3 (Fig. 5A). Interestingly, cells treated with DP or CI994 could overcome this downregulation, and LC3 was found to be localized as punctate structures in the cells, and the number of punctae was higher in cells treated with both drugs (Fig. 5A).

**FIG 6:**
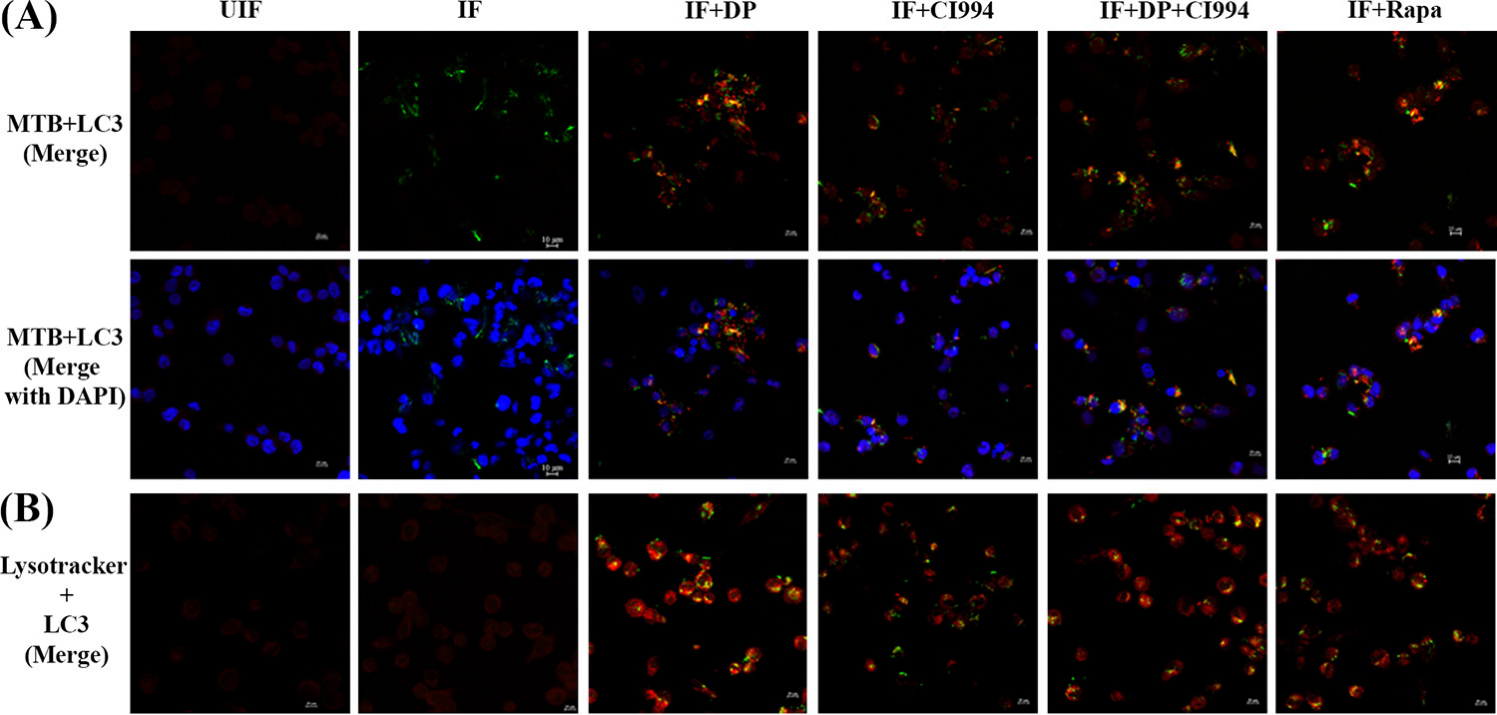
DP and CI994 induce autophagic clearance of bacteria by enhancing the colocalization M. tuberculosis-LC3 and LC3-lysosome in human macrophages. THP-1-derived macrophages were infected with the virulent strain of M. tuberculosis expressing GFP (for 4 h) and treated with DP, CI994, and rapamycin (Rapa). (A) Distribution of colocalization of GFP-expressing M. tuberculosis and LC3 (red) in macrophages at 24 hpi. (B) Macrophages were immunostained for LC3 (green) and LysoTracker Red to detect LC3 and lysosome-containing cells.

LC3 is usually located in the cytoplasm of cells as LC3-I, a nonlipidated form of the former. After induction of autophagy, phosphatidylethanolamine binds covalently to LC3 to form LC3-II, which is associated with the membrane of autophagosomes (18). We analyzed the LC3-I to LC3-II conversion by Western blot analysis (Fig. 5B and C). Enhanced formation of LC3-II was observed in M. tuberculosis*-*infected macrophages treated with DP and CI994. Subsequently, employing fluorescence microscopy, we tracked the association of green fluorescent protein (GFP)-expressing M. tuberculosis with autophagosomes in infected macrophages upon treatment with the inhibitors. DP and CI994 treatment caused an increase in the number of cells with LC3 punctae and showed an increase in the colocalization of GFP-expressing M. tuberculosis with LC3 autophagosomes (Fig. 6A; Fig. S6B) compared to the untreated cells. As autophagy progresses, autophagosomes combine with lysosomes to form autolysosomes that carry LC3 and various lysosome-associated components. To find whether DP/CI994 treatment and IL-12p40 restoration affect autolysosome formation, we analyzed the colocalization of endogenous LC3 and lysosomes by immunostaining with LysoTracker followed by fluorescence microscopy. LC3 vesicles were found to be colocalized with lysosomes as clearly seen in Fig. 6B. Treatment of uninfected normal macrophages with DP and CI994 did not cause an increase in the levels of LC3, and cells containing lysosomes were not detected (Fig. S6C).

### Treatment of infected macrophages with IL-12p40 enhances colocalization of M. tuberculosis and LC3.

**FIG 7:**
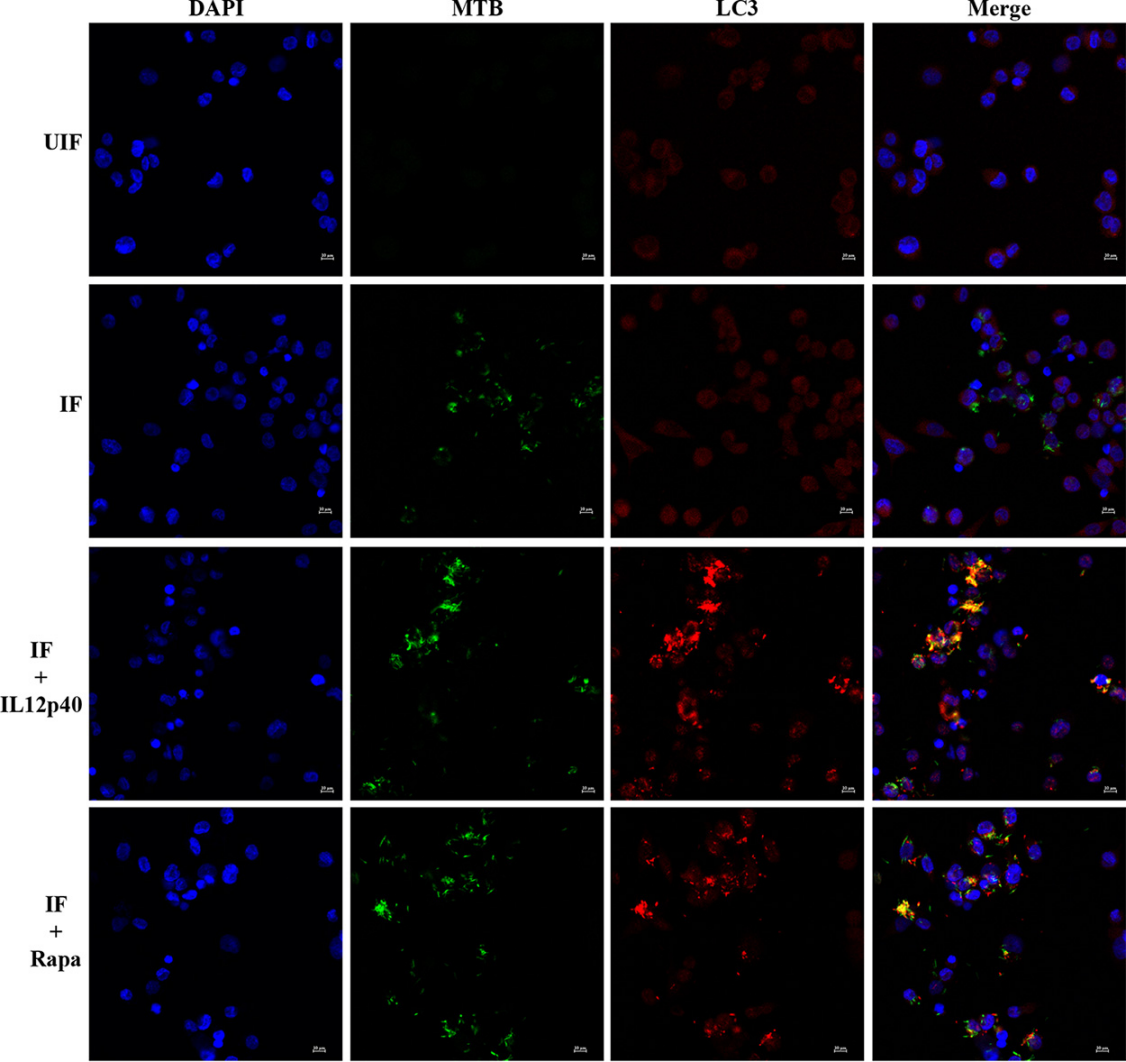
Treatment of M. tuberculosis infected-macrophages with IL-12p40 induces autophagy by enhancing the expression of LC3 and M. tuberculosis-LC3 colocalization. Colocalization of GFP-expressing M. tuberculosis and LC3 (red) in macrophages at 24 hpi.

We sought to reconfirm whether autophagy activation is mediated by the IL-12p40 protein in macrophages infected with M. tuberculosis. For this, mycobacteria-infected cells were treated with recombinant human IL-12p40 (Abcam, USA) for 24 h, after which we examined whether IL-12p40 treatment could enhance the levels of LC3. IL-12p40 treatment caused an increase in the number of cells with LC3 punctae and showed an increase in the colocalization of GFP-expressing M. tuberculosis with LC3 autophagosomes (Fig. 7).

### IL-12p40-mediated activation of autophagy depends on the JAK-STAT pathway.

**FIG 8:**
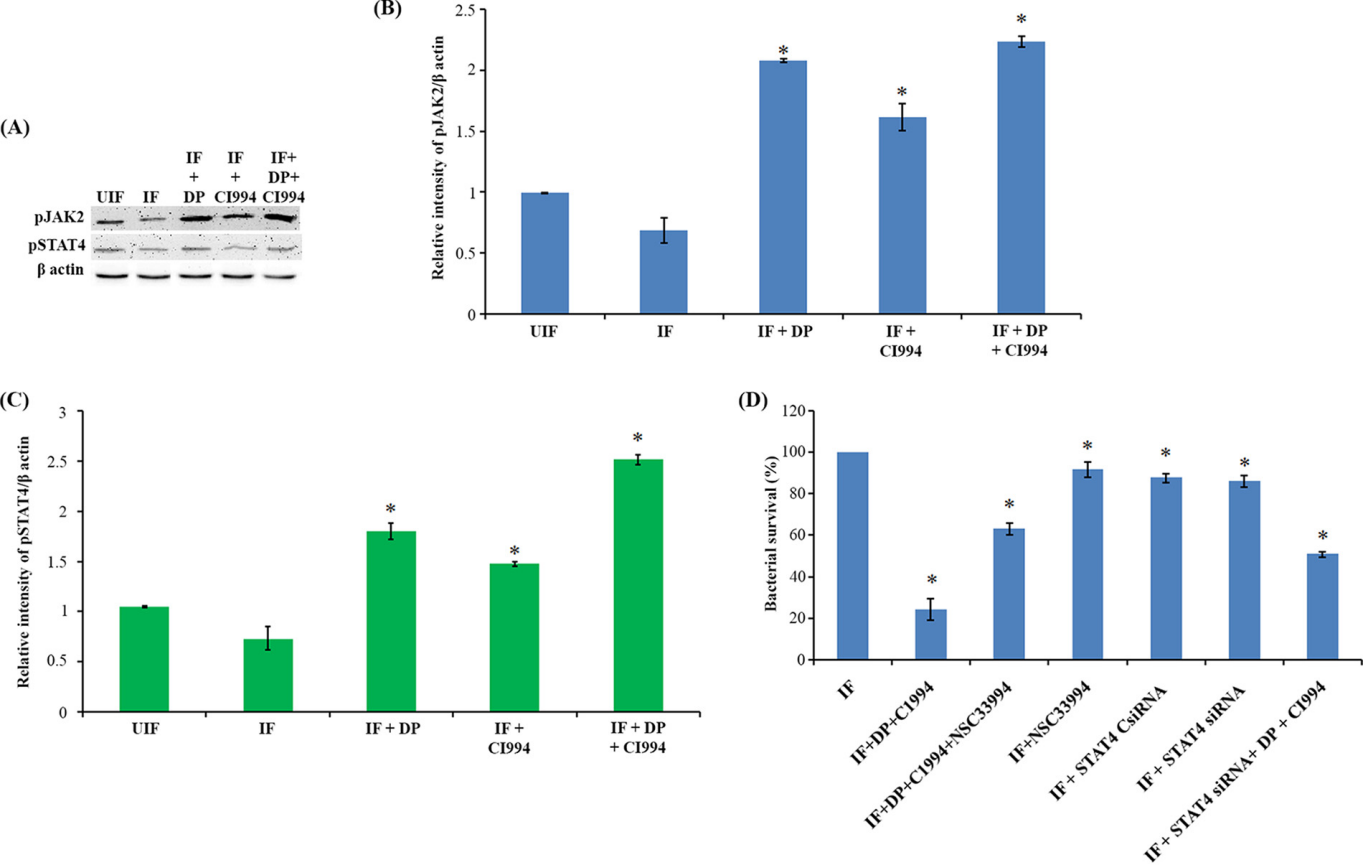
IL-12p40-mediated activation of autophagy depends on the JAK-STAT pathway. (A) Status of pJAK2 and pSTAT4 in infected macrophages after treatment with DP and CI994. (B and C) Densitometric analysis of pJAK2 (B) and STAT4 (C) bands normalized with that of β-actin. Each value represents mean ± SD from triplicate measurements. *, band intensity is significantly different from infected (IF) samples individually treated with inhibitors, respectively; *P* ≤ 0.05. (D) Infected THP-1-derived macrophage cells were treated with JAK2 inhibitor NSC33994 along with DP and CI994. THP-1-derived macrophages deficient in STAT4 were treated with DP and CI994. Intracellular bacterial viability was determined based on the number of CFU. Isolation of bacilli from the macrophages was carried out at 24 h. The number of viable bacilli in each of the plate was assayed by plating lysed macrophages on 7H10 agar plates and incubating the plates at 37°C for 3 weeks and counting the CFU. Each value represents mean ± SD from triplicate measurements. *, survival is significantly different from infected (IF) sample; *P* ≤ 0.05.

To find the role of pJAK2 and pSTAT4 in the activation of IL-12p40-mediated induction of autophagy, we assayed their levels in macrophage upon M. tuberculosis infection by Western blotting (Fig. 8A to C). We observed a significant increase in the levels of phosphorylation of JAK2 and STAT4 upon treatment of infected macrophages with DP and CI994. Treatment of infected cells with a specific JAK2 inhibitor, NSC33994, confirmed that autophagy is induced via a JAK2-dependent pathway (Fig. 8D). Therefore, we tested the effect of NSC33994 on the viability of intracellular M. tuberculosis in DP and CI994-treated macrophages. Interestingly, inhibition of the JAK2 signaling pathway by NSC33994 was found to be counteractive to the treatment with DP/CI994 and led to the restoration of the viability of intracellular M. tuberculosis (Fig. 8D). To investigate the role of STAT4, we knocked down *STAT4* in differentiated macrophages (Fig. S7 at https://rgcb.res.in/documents/publication/drajay/mSPhere00036-21%20supplemental%20file%20RGCB.pdf) and infected them with M. tuberculosis. When we treated these infected macrophages with DP and CI994, we did not observe any significant reduction in the number of intracellular M. tuberculosis, confirming the role of STAT4 in inducing IL-12p40-mediated autophagy (Fig. 8D).

## DISCUSSION

Earlier, our laboratory had demonstrated that levels of HDAC1, a ubiquitous suppressor of gene expression, increase in macrophages infected with M. tuberculosis, and it hypoacetylates histone H3 at the promoter of *IL-12B* gene (10). The product of this gene plays a crucial role in initiating Th1 responses after infection with intracellular pathogens. HDAC1 is usually found in multiprotein corepressor complexes containing Sin3a, nucleosome-remodeling (NuRD), and CoREST, which are recruited to the promoter region of different genes by transcription factors like Sp1, Sp3, p53, NF-κB, and YY1 (19). The SIN3A/HDAC complex is recognized as a global transcriptional corepressor. The Sin3a protein provides a platform for multiple protein interactions with its four-paired amphipathic α-helix motif (20). HDAC1 forms a repressor complex, and its function is regulated by the proteins it associates with in the complex, which determine the enzymatic activity and recruitment to specific DNA sequences (21). Phosphorylation of HDACs is one of the most widely studied posttranslational modifications, and the functional role of HDAC1, its cellular localization, and interaction with other proteins are regulated by this modification (22). The identity and role of proteins associated with pHDAC1 during M. tuberculosis infection, and their role in the progress of infection and pathogenesis, are not known. Time course analysis revealed that HDAC1 phosphorylation is high at 24 hpi, and we presume that this modification contributes to its ability to bind to its associated proteins. In the present study, mass spectrometric analysis of immunoprecipitated pHDAC1-interacting proteins revealed the presence of an uncommon protein in the complex. We found that in addition to HDAC1 and Sin3a, the repressor complex in M. tuberculosis-infected macrophages contained the transcriptional repressor ZBTB25. We substantiated the association of ZBTB25 with HDAC1 and Sin3a by reciprocal coimmunoprecipitation and Western blot analyses.

ZBTB25 is a transcriptional repressor, and the exact physiological function of the ZBTB family is not well understood; a few ZBTB proteins have been reported to participate in tumor progression and chromatin remodeling and have a repressive role in the differentiation and activation of T cells (23, 24). It has been shown that transcriptional repressors participate in the progression of a variety of diseases (25–27). In human lung adenocarcinoma epithelial cells infected with influenza virus, ZBTB25 was found to interact with the viral RNA-dependent RNA polymerase (RdRp) and to enhance its transcription. In addition, ZBTB25 suppresses interferon production, further enhancing influenza viral replication (28). Nonetheless, to the best of our information, any functional role of transcriptional repressors of the ZBTB family involved in bacterial infection has not been reported. ZBTB25 contains a DNA-binding zinc finger motif at the C terminus and a protein-binding BTB/POZ domain at the N terminus. Proteins with these two conserved domains are categorized as POZ and Krüppel (POK) proteins, also called the zinc finger and BTB domain (ZBTB) family (29). Genomes of mouse and human code for more than 40 POK type proteins, which include promyelocytic leukemia zinc finger proteins, ZBTB2, ZBTB7, ZBTB16, and ZBTB20 (30–33).

The present study showed that ZBTB25-associated HDAC1 silencing complex is recruited to the *IL-12B* promoter and suppresses expression of the *IL-12B* gene, thereby downregulating the host’s Th1 immune responses against intracellular bacterial pathogens. This generates a beneficial environment for M. tuberculosis inside the macrophages. Presumably, the protein-binding domain of ZBTB25 interacts with HDAC1, and the DNA-binding domain binds to the *IL-12B* promoter sequences, respectively, since this protein has both DNA-binding and protein-binding domains (29). We confirmed this interaction by *in silico* docking, immunoprecipitation and chromatin immunoprecipitation, and confocal microscopy. In addition, we found that DP, inhibitor of ZBTB25, blocked the recruitment of HDAC1 silencing complex to the *IL-12B* promoter. DP is a zinc ejector that abrogates the functionality of the zinc finger domain (34, 35) and has been reported to inactivate the zinc finger nucleocapsid of HIV-1 (36). Labile zinc finger core is the typical target for zinc-ejecting drugs. Some zinc finger cores are vulnerable to electrophilic agents and can function as promising drug targets for cancer and viral therapeutics, including SARS-CoV-2 (37). While not all zinc finger proteins have a labile zinc finger core, ZBTB25 is reported to have one (28). We report that DP is an effective zinc ejector drug against ZBTB25, and its IC_50_ value (4.61 μM) is less than that of other zinc ejector drugs like DS and 2-NB. Lee et al. (35) have reported that DP can inhibit various zinc finger proteins, but the inhibition is specifically dependent on the concentration of DP used; the IC_50_ value of DP of 4.61 μM toward ZBTB25 is lower than that of many other zinc finger proteins reported (ubiquitin-like with PHD and ring finger domains 1 [UHRF1], 7.46 μM; BHC80, 8.14 μM; TRAF6, 8.7 μM; and DNMT3L, 5.13 μM).

Since CI994 is a class I HDAC inhibitor, we assessed the specificity of inhibition of the same toward HDAC1, HDAC2, and HDAC3. We found that it most effectively inhibited HDAC1 (at 0.52 ± 0.03 μM) followed by HDAC2 and HDAC3 (1.09 ± 0.07 μM and 1.54 ± 0.09 μM, respectively). This finding is consistent with a previous study by Zhou et al. (38), who determined the inhibitory concentrations of CI994 toward HDAC1, HDAC2, and HDAC3 as 0.41 μM, 0.9 μM, and 1.2 μM, respectively. HDAC8 is inhibited by CI994 at concentrations above 100 μM (39). Our studies in macrophages infected with M. tuberculosis suggested that 20 μM DP is required to prevent the recruitment of ZBTB25 to the *IL-12B* promoter. Similar to DP, the recruitment of HDAC1 to the *IL-12B* promoter was found to be reduced significantly at ≥15 μM concentration.

We further found that knocking down of ZBTB25 disrupts the recruitment of HDAC1 to the *IL-12B* promoter and subsequently derepresses the gene expression. Thus, ZBTB25 plays a crucial role in the recruitment of HDAC1 silencing complex to the *IL-12B* promoter in macrophages infected with M. tuberculosis *in vitro*. Treatment with the combination of DP and CI994 significantly reduced the amount of viable M. tuberculosis in infected macrophages. Phenylbutyrate, a pan-HDAC inhibitor, has been shown to induce *CAMP/LL-37* gene expression in macrophages and inhibit M. tuberculosis growth (40).

The results from our study show that the inhibition of ZBTB25 and HDAC1 increases the levels of IL-12p40 (a subunit of the cytokine IL-12) in macrophages infected with M. tuberculosis. Since IL-12 has been shown to be an activator of autophagy (41), we investigated if autophagy can be induced in THP-1-derived macrophages by treating them with DP and CI994. Autophagy is one of the homeostatic machineries mediated through lysosomes, and it acts as an inherent defense mechanism against M. tuberculosis infection. Host innate immunity uses autophagy to clear M. tuberculosis and other intracellular pathogens (40, 42, 43). Macrophages phagocytose and sequester M. tuberculosis in phagosomes, which can then fuse with lysosomes for bacterial degradation. However, Gutierrez et al. showed that M. tuberculosis has the ability to survive and grow inside macrophages by preventing phagosome-lysosome fusion (44). They showed that starvation or treatment of macrophages with rapamycin increases M. tuberculosis colocalization with LC3 and Beclin 1 and transfers M. tuberculosis to phagolysosomes for subsequent autophagy. Autophagy induction can be harnessed toward stimulating macrophage defense against M. tuberculosis (45). It has been shown that antiprotozoal drug nitazoxanide and its active metabolite tizoxanide strongly stimulate autophagy and inhibit signaling by mechanistic target of rapamycin complex 1 (mTORC1), a major negative regulator of autophagy, and inhibit M. tuberculosis proliferation in infected human THP-1 cells and peripheral monocytes (46). Antimycobacterial drugs like isoniazid and pyrazinamide have been shown to induce autophagy and phagosomal maturation in M. tuberculosis-infected host cells (47). Loperamide and the cholesterol-lowering drug statin were also reported to induce autophagy and phagosomal maturation and reduce intracellular growth of M. tuberculosis in infected macrophages, suggesting that host autophagy plays a key role in host immune responses during drug administration against TB (48, 49). We here showed that treatment of THP-1-derived macrophages with DP and CI994 restores the levels of IL-12p40 and leads to the activation of major autophagic markers, enhances intracellular LC3 distribution, and causes colocalization of M. tuberculosis with LC3. Moreover, treatment of M. tuberculosis-infected macrophages with IL-12p40 enhanced the levels of LC3 and colocalization of M. tuberculosis and LC3 in the autophagosome, suggesting a critical role of IL-12p40 in the induction of autophagy. These observations suggest that treatment of macrophage with DP and CI994 could be an effective antimicrobial strategy against intracellular M. tuberculosis by inducing autophagy. Interestingly, Jung et al. (50) showed that IL-12 is involved in antimicrobial action by activating the fusion of phagosome and lysosome via interferon gamma (IFN-γ). Although IL-12-induced autophagy has been reported in breast cancer cells, human macrophages, and lung epithelial cells (41, 51), the precise function of IL-12 in inducing this process is not yet clearly understood.

**FIG 9:**
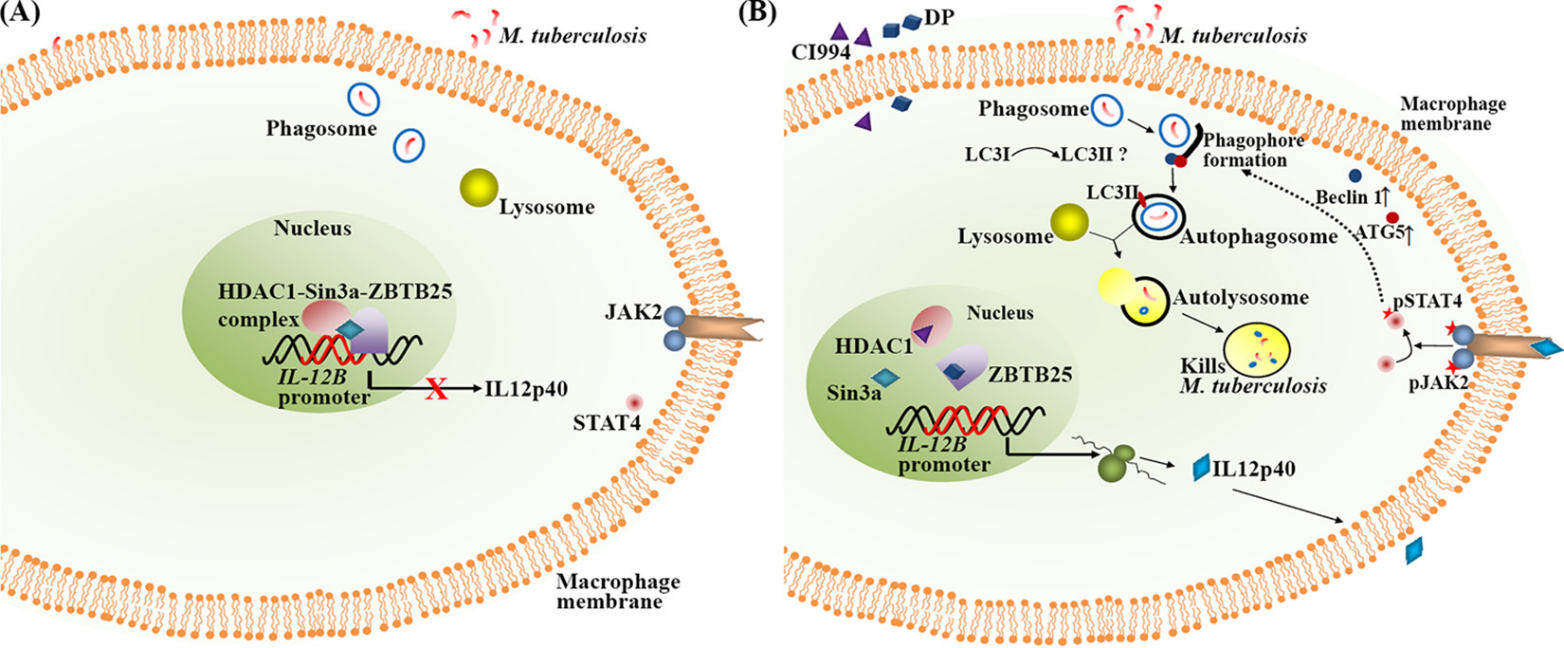
Proposed model for the induction of autophagy and killing of M. tuberculosis mediated by the inhibition of ZBTB25 and HDAC1. (A) Status of *IL-12B* expression upon M. tuberculosis infection. HDAC1-Sin3a-ZBTB25 complex suppresses *IL-12B* expression in macrophages. (B) Treatment of infected macrophages with inhibitors DP and CI994 derepresses *IL-12B* expression and induces autophagy and killing of M. tuberculosis through the JAK2/STAT4 pathway. Stars indicate phosphorylated forms of proteins.

IL-12 activates tyrosine phosphorylation of Janus family tyrosine kinases JAK2 and Tyk2, suggesting the involvement of these kinases in the biochemical response to IL-12. The substrates for the JAKs are the family of latent cytoplasmic transcription factors termed STATs (52). The presence of the phosphotyrosine dimer in STATs is vital in target gene activation (53). Bacon et al. have demonstrated that IL-12 induces tyrosine phosphorylation of STAT4 in lymphocytes (54). Thus, IL-12 is the major cytokine that can activate STAT4, synthesized predominantly by dendritic cells, macrophages, and human B-lymphoblastoid cells (55). To study the mechanism by which IL-12p40 activates autophagy, we evaluated the status of JAK2 and STAT4 in the macrophages infected with M. tuberculosis. Enhanced phosphorylation of JAK2/STAT4 was observed in infected macrophages upon DP/CI994 treatment, which suggests the involvement of IL-12p40 in the phosphorylation of JAK2 and STAT4. Inhibition of JAK2 by specific inhibitor NSC33994, and knocking down of *STAT4*, also led to an increase in intracellular survival of M. tuberculosis through inhibition of DP/CI994-induced autophagy. Taken together, these results clearly demonstrate that DP and CI994-mediated restoration of IL-12p40 levels induces autophagy by phosphorylating JAK2 and STAT4 that eventually kills intracellular M. tuberculosis (Fig. 9). However, it remains to be seen whether the effect is brought about by IL-12p40 *per se* or by IL-12.

Host-directed therapy (HDT) is a novel method suggested for the treatment of infectious diseases, where host response is enhanced by treatment with different drugs, with or without antimicrobials, to achieve a better outcome. HDT drugs target autophagy, vitamin D pathway, and anti-inflammatory responses (56, 57). Jayaswal et al. (58) highlighted the importance of host factors that regulate intracellular survival of M. tuberculosis through an siRNA screen in J774.1 murine macrophage cells. They could identify host factors such as TGFβRI and CSNK1, and their inhibition substantially reduced the number of intracellular M. tuberculosis in J774.1 cells as well as in primary murine macrophages. Pharmacological inhibition of the AKT/mTOR pathway in host reduces intracellular growth of M. tuberculosis in human PBMCs and *in vivo* in a model of murine tuberculosis (59). Targeted inhibition of NF-κB activation decreases viability of intracellular M. tuberculosis in human macrophages (60). HDAC6 inhibitor tubastatin A has been shown to reduce M. tuberculosis survival in an *in vivo* murine model (61). The pan-HDAC inhibitor trichostatin A and class IIa HDAC inhibitor TMP195 have been shown to be very effective in reducing M. tuberculosis infection in primary human macrophages and a Mycobacterium marinum-zebrafish infection model (62). Rao et al. (63) evaluated the possibility of HDAC inhibitors valproic acid and suberoylanilide hydroxamic acid to enhance the activity of first-line anti-TB drugs to inhibit the growth of intracellular M. tuberculosis.

We carried out this study to identify host proteins other than HDACs that could also be used as a target for HDT. As our proteomic analysis revealed that ZBTB25 is an integral component of the HDAC1/Sin3a repressor complex that repressed expression of *IL-12B*, we tested if its inhibition could bring about an effect similar to that caused by the inhibition of HDAC1. Our inhibition and knockdown studies demonstrated that ZBTB25 is an equally worthy target, as its inhibition resulted in the derepression of *IL-12B* expression. DP was found to be more effective in killing intracellular M. tuberculosis than CI994 (65% and 53% killing, respectively). However, the combination of DP and CI994 did not show a cumulative effect in killing intracellular bacteria and was only slightly better (72% killing) than DP alone. These observations suggest that ZBTB25, like HDAC1, is an attractive target for a possible host-directed therapeutic intervention to treat tuberculosis.

## MATERIALS AND METHODS

### Bacterial strains.

M. tuberculosis strains H37Rv and M. tuberculosis H37Rv expressing GFP constitutively were used in the current study, and these M. tuberculosis strains were handled in a biosafety level 3 (BSL3) facility. Middlebrook 7H9 broth (Difco, USA) supplemented with 10% oleic acid-albumin-dextrose-catalase (OADC) (Becton, Dickinson, USA), 0.4% glycerol, and 0.05% Tween 80 was used to grow the M. tuberculosis strains. Bacteria in the mid-log phase were centrifuged, washed, and resuspended in RPMI medium containing fetal bovine serum (FBS) (10%). The suspension was dispersed by aspirating several times and vortexed until no bacterial clumps were visible and allowed to stand for 5 min. The upper half of the suspension was collected (absorbance of 0.15 at 600 nm corresponds to ∼3 × 10^8^ bacteria) to perform various experiments.

### Resazurin microtiter assay.

Resazurin microtiter assay (REMA) was performed as described by Martin et al. (64). In short, the turbidity of M. tuberculosis culture was adjusted to 0.15 units at 600 nm and diluted 20 times. The diluted bacterial suspension was added to the wells of a 96-well microtiter plate (Nunc; Thermo Fisher Scientific, Denmark) containing 100 μl of dithiopyridine (DP; Sigma-Aldrich, USA) and CI994 (Sigma-Aldrich, USA) or rifampicin (Sigma-Aldrich, USA) solutions. The microtiter plates were incubated for 7 days with appropriate controls. After incubation, the minimum inhibitory concentration (MIC) was determined by adding resazurin (30 μl of 0.02% aqueous solution) to each well. A blue color of the culture would indicate inhibition of bacterial growth, whereas a pink color would indicate the presence of viable bacteria.

### Cytotoxicity assay by MTT.

The MTT assay was performed as described by Mosmann (65). Briefly, 10,000 cells were seeded per well in a 96-well plate. They were treated with DP and CI994 and incubated for 48 h at 37°C in the presence of 5% CO_2_. Ten microliters of MTT [3-(4,5-dimethylthiazole-2-yl)-2,5-diphenyl tetrazolium bromide] dye (5 mg/ml) were added to each well, and after 4 h, the reaction was terminated by adding 200 μl of dimethyl sulfoxide (DMSO) to dissolve the formazan crystals. This was read at 540 nm on an ELISA reader (Bio-Rad, USA).

### Zinc ejection assay.

The release of zinc ions from ZBTB25 protein was detected by the fluorescence emitted from the zinc-specific fluorophore FluoZin-3 (Life Technologies, USA). ZBTB25 protein (Novus Biologicals, USA) was treated with various concentrations of DP, disulfiram (DS; Sigma-Aldrich, USA), 2-nitrobenzoic acid (2NB; Sigma-Aldrich, USA), and FluoZin-3 (5 μM) at room temperature. Fluorescence emission was monitored at an excitation wavelength of 494 nm and emission wavelength of 516 nm for 20 min.

### HDAC inhibition assay.

The assay was performed using HDAC inhibition assay kit (Cayman Chemicals, USA), according to the manufacturer's protocol. Ten microliters of CI994 (different concentrations) or positive control (Trichostatin A, 10 μl) were added to the mixture of assay buffer (140 μl) and respective HDACs (10 μl). The reaction was initiated by adding HDAC substrate (10 μl) and incubated for 30 min at 37°C with mild shaking (75 rpm). After the incubation, the developer solution (40 μl) was added to each well and incubated at room temperature for 15 min. The fluorescence developed was measured using an excitation wavelength of 340 to 360 nm and an emission wavelength of 440 to 465 nm. The percentage of HDAC inhibition was calculated as follows:

$$\text{HDAC inhibition}\;(\%)=\left(\frac{\text{initial activity}-\text{activity after inhibition}}{\text{initial activity}}\right)\times 100$$

### Infection of THP-1-derived macrophage cells with M. tuberculosis H37Rv.

RPMI 1640 medium containing FBS (10%), l-glutamine (2 mM), HEPES (25 mM), and Na_2_CO_3_ (1.5 g/liter) was used to culture THP-1 cells at 37°C and in the presence of 5% CO_2_. THP-1-derived macrophages were treated with phorbitol 12-myristate 13-acetate (PMA) (20 ng/ml) for 24 h to differentiate them into macrophages. The differentiated cells were mixed with M. tuberculosis at 20:1 multiplicity of infection (MOI) and were incubated for 4 h at 37°C in the presence of 5% CO_2_ to facilitate phagocytosis. Cells were then washed four times with complete RPMI containing gentamicin (10 μg/ml) to remove extracellular bacilli, and fresh medium was added to the cells. For experiments in which inhibitors were used, specific inhibitors were added after phagocytosis. Final concentrations of the inhibitors were 20 μM/ml DP, 15 μM/ml CI994, and 100 nM/ml rapamycin.

Apart from THP-1-derived macrophage cells, primary macrophages obtained from peripheral blood mononuclear cells (PBMC; Sigma) were also used in the study. PBMCs were incubated at 37°C in the presence of 5% CO_2_ for 2 h. The nonattached cells were removed by washing thrice with RPMI 1640. The remaining attached cells were grown in RPMI supplemented with FBS (10%), incubated for 4 days to differentiate them into macrophages, and infected with M. tuberculosis.

### Transfection of macrophages with siRNA and infection with M. tuberculosis.

THP-1 cells grown in Opti-MEM medium (Invitrogen, Carlsbad, USA) were differentiated and transfected (HiPerFect transfection reagent; Qiagen, Valencia, CA, USA) with 60 pmol of siRNA (Santa Cruz Biotechnologies, USA) for 36 h. Transfected cells were infected with M. tuberculosis H37Rv at an MOI of 20:1. Cells transfected with scrambled siRNA served as control.

### Confocal microscopy.

Differentiated THP-1 cells were infected with M. tuberculosis H37Rv and M. tuberculosis H37Rv expressing GFP and were incubated for 4 h to facilitate phagocytosis. Cells were then washed four times with complete RPMI containing gentamicin (10 μg/ml) to remove extracellular bacilli, and fresh medium was added to the cells. Cells were fixed with paraformaldehyde (4%), incubated with methanol for 20 min at −20°C, and then blocked with phosphate-buffered saline (PBS) containing bovine serum albumin (BSA) (3%, wt/vol), and Triton X-100 (0.3%, vol/vol). The cells were incubated for 1 h at room temperature with anti-rabbit or anti-mouse primary antibodies against pHDAC1 (EMD Millipore, USA), ZBTB25 (Sigma-Aldrich, USA), LC3 (Santa Cruz Biotechnology, USA), ATG5 (Santa Cruz Biotechnology, USA), and BECN1 (Santa Cruz Biotechnology, USA), washed, and incubated with fluorescein isothiocyanate (FITC)-conjugated anti-rabbit secondary antibody (Sigma-Aldrich, USA) or Alexa 488/Alexa 592 anti-rabbit or anti-mouse secondary antibody (Thermo Fisher, USA) for 1 h at room temperature. Cells were counterstained with 4′,6-diamidino-2-phenylindole (DAPI) for DNA. To detect lysosomes, cells were incubated with LysoTracker Red (Thermo Fisher Scientific, USA). Rapamycin (Sigma-Aldrich, USA) was used as a positive control for autophagy induction.

### Immunoprecipitation.

Differentiated THP-1 cells (infected and uninfected) were harvested, washed with PBS, and then extracted with immunoprecipitation (IP) lysis buffer for 30 min at 4°C. Specific primary antibodies or rabbit IgG was added to the lysates of infected and uninfected cells and incubated overnight at 4°C. Protein-A/G-coated Sepharose beads were added to the antibody-protein mixture and incubated for 2 h at 4°C. Beads were washed five times with lysis buffer, and the proteins were eluted in 0.2 M glycine and precipitated with trichloroacetic acid (TCA; 10%) in cold acetone (wt/vol). The precipitate was dissolved in 50 mM ammonium carbonate and was subjected to LC-MS/MS analysis. For Western blotting, the immune complexes were eluted in SDS-PAGE sample buffer (0.0005% bromophenol blue, 10% glycerol, 2% SDS, and 63 mM Tris-HCl, pH 6.8).

### Chromatin immunoprecipitation.

Chromatin immunoprecipitation (ChIP) was carried out based on the manufacturer's protocol (Abcam, UK). In brief, the infected and uninfected macrophages were treated with 4% formaldehyde in PBS for 10 min and washed with PBS. Cells were lysed with ChIP lysis buffer, and the chromatin was sheared by sonication (Bioruptor; Diagenode, Belgium) with pulse conditions 30 s on, 30 s off for 25 cycles at 4°C followed by centrifugation (14,000 rpm for 15 min at 4°C). Antibodies against HDAC1, ZBTB25, Sin3a, or IgG were used to immunoprecipitate the chromatin fragments in the supernatant by overnight incubation using protein A Sepharose beads (Abcam, UK) at 4°C. The beads were washed twice with wash buffer, DNA was eluted off the beads, and the purified DNA was subjected to PCR. Primers used for PCR amplification of specific gene promoters are listed in Table S2 at https://rgcb.res.in/documents/publication/drajay/mSPhere00036-21%20supplemental%20file%20RGCB.pdf.

### Molecular docking.

To infer the interaction between HDAC1 and ZBTB25, we performed docking studies by utilizing AutoDock 4 software (66).

### Western blotting.

Nuclear extracts (using NE-PER nuclear extraction kit; Thermo Fisher Scientific, USA) and whole-cell lysates (using radioimmunoprecipitation assay [RIPA] buffer; Sigma, USA) were prepared from M. tuberculosis-infected macrophages (MOI of 20:1). Proteins were separated on SDS-polyacrylamide (12%) gels, transferred to Immobilon-P polyvinylidene fluoride membranes (Millipore, Billerica, MA, USA), and probed with protein-specific primary antibody. Horseradish peroxidase (HRP)-conjugated anti-rabbit secondary antibody was added to visualize the proteins by chemiluminescence, and image quantification was carried out using ImageJ software (NIH, USA). Relative intensity of specific proteins was plotted with respect to the band intensity of histone H3 or actin.

### Quantitative real-time PCR.

The total RNA was isolated from macrophages (infected and uninfected) using NucleoSpin RNA extraction kit (Macherey-Nagel, Germany) according to the manufacturer's protocol. RNA (1 μg) was converted to cDNA using the GoScript reverse transcription system (Promega, Madison, WI). The cDNA synthesized was used for qPCR using the iQ SYBR green SuperMix (Bio-Rad Laboratories, Hercules, CA) on an iCycler iQ real-time PCR detection system (Bio-Rad, USA). Fold changes in gene expression levels were calculated and were normalized to human actin. The list of quantitative real-time PCR (qPCR) primers (from Sigma-Aldrich, USA) is shown in Table S2.

### ELISA.

The IL-12p40 in cell supernatants of infected and uninfected macrophages was quantified using BD OptEIA Human IL-12(p40) ELISA kit (Abcam, UK) as per the manufacturer's directives.

### Intracellular survival assays for M. tuberculosis.

The THP-1-derived macrophage cells were infected with M. tuberculosis as described elsewhere. After 4 h, cells were washed four times with complete RPMI medium containing gentamicin (10 μg/ml) to remove extracellular bacilli, and fresh medium was added. Cells were harvested at 24 h after infection. The monolayers of macrophages were washed with PBS and lysed with SDS (0.06%) in 7H9 medium, and the lysate was spread on 7H10 agar plates and incubated at 37°C to determine the intracellular CFU. After 3 weeks, the bacterial colonies on the plates were counted. The experiment was performed in triplicates.

### Statistical analysis.

The data were expressed as mean values with their standard deviations. The results were analyzed by nonparametric analysis of variance (ANOVA). A *P* value of <0.05 was considered significant.

## References

[1] Zumla A, Rao M, Parida SK, Keshavjee S, Cassell G, Wallis R, Axelsson-Robertsson R, Doherty M, Andersson J, Maeurer M. 2015. Inflammation and tuberculosis: host-directed therapies. J Intern Med 277:373–387. doi:10.1111/joim.12256. 24717092

[2] Kaufmann SHE, Dorhoi A, Hotchkiss RS, Bartenschlager R. 2018. Host-directed therapies for bacterial and viral infections. Nat Rev Drug Discov 17:35–56. doi:10.1038/nrd.2017.162. 28935918 PMC7097079

[3] Deretic V, Levine B. 2009. Autophagy, immunity, and microbial adaptations. Cell Host Microbe 5:527–549. doi:10.1016/j.chom.2009.05.016. 19527881 PMC2720763

[4] Barry CE, Boshoff HI, Dartois V, Dick T, Ehrt S, Flynn J, Schnappinger D, Wilkinson RJ, Young D. 2009. The spectrum of latent tuberculosis: rethinking the biology and intervention strategies. Nat Rev Microbiol 7:845–855. doi:10.1038/nrmicro2236. 19855401 PMC4144869

[5] Jayaraman P, Sada-Ovalle I, Nishimura T, Anderson AC, Kuchroo VK, Remold HG, Behar SM. 2013. IL-1β promotes antimicrobial immunity in macrophages by regulating TNFR signaling and caspase-3 activation. J Immunol 190:4196–4204. doi:10.4049/jimmunol.1202688. 23487424 PMC3622150

[6] Law KF, Jagirdar J, Weiden MD, Bodkin M, Rom WN. 1996. Tuberculosis in HIV-positive patients: cellular response and immune activation in the lung. Am J Respir Crit Care Med 153:1377–1384. doi:10.1164/ajrccm.153.4.8616569. 8616569

[7] Robinson CM, Nau GJ. 2008. Interleukin‐12 and interleukin‐27 regulate macrophage control of Mycobacterium tuberculosis. J Infect Dis 198:359–366. doi:10.1086/589774. 18557702 PMC2761687

[8] Salgame P. 2005. Host innate and Th1 responses and the bacterial factors that control Mycobacterium tuberculosis infection. Curr Opin Immunol 17:374–380. doi:10.1016/j.coi.2005.06.006. 15963709

[9] Kathirvel M, Mahadevan S. 2016. The role of epigenetics in tuberculosis infection. Epigenomics 8:537–549. doi:10.2217/epi.16.1. 27035266

[10] Chandran A, Antony C, Jose L, Mundayoor S, Natarajan K, Kumar RA. 2015. Mycobacterium tuberculosis infection induces HDAC1-mediated suppression of IL-12B gene expression in macrophages. Front Cell Infect Microbiol 5:90. doi:10.3389/fcimb.2015.00090. 26697414 PMC4667035

[11] Zhou J, Snyder AR, Lieberman PM. 2009. Epstein-Barr virus episome stability is coupled to a delay in replication timing. J Virol 83:2154–2162. doi:10.1128/JVI.02115-08. 19073720 PMC2643711

[12] Knight JS, Lan K, Subramanian C, Robertson ES. 2003. Epstein-Barr virus nuclear antigen 3C recruits histone deacetylase activity and associates with the corepressors mSin3A and NCoR in human B-cell lines. J Virol 77:4261–4272. doi:10.1128/jvi.77.7.4261-4272.2003. 12634383 PMC150657

[13] Terhune SS, Moorman NJ, Cristea IM, Savaryn JP, Cuevas-Bennett C, Rout MP, Chait BT, Shenk T. 2010. Human cytomegalovirus UL29/28 protein interacts with components of the NuRD complex which promote accumulation of immediate-early RNA. PLoS Pathog 6:e1000965. doi:10.1371/journal.ppat.1000965. 20585571 PMC2891856

[14] Bandyopadhaya A, Tsurumi A, Rahme LG. 2017. NF-κBp50 and HDAC1 interaction is implicated in the host tolerance to infection mediated by the bacterial quorum sensing signal 2-aminoacetophenone. Front Microbiol 8:1211. doi:10.3389/fmicb.2017.01211. 28713342 PMC5492500

[15] Rennoll-Bankert KE, Garcia-Garcia JC, Sinclair SH, Dumler JS. 2015. Chromatin-bound bacterial effector ankyrin A recruits histone deacetylase 1 and modifies host gene expression. Cell Microbiol 17:1640–1652. doi:10.1111/cmi.12461. 25996657 PMC5845759

[16] Chang CC, Ye BH, Chaganti RS, Dalla-Favera R. 1996. BCL-6, a POZ/zinc-finger protein, is a sequence-specific transcriptional repressor. Proc Natl Acad Sci U S A 93:6947–6952. doi:10.1073/pnas.93.14.6947. 8692924 PMC38914

[17] Beaulieu AM, Sant'Angelo DB. 2011. The BTB-ZF family of transcription factors: key regulators of lineage commitment and effector function development in the immune system. J Immunol 187:2841–2847. doi:10.4049/jimmunol.1004006. 21900183 PMC3170133

[18] Runwal G, Stamatakou E, Siddiqi FH, Puri C, Zhu Y, Rubinsztein DC. 2019. LC3-positive structures are prominent in autophagy-deficient cells. Sci Rep 9:10147. doi:10.1038/s41598-019-46657-z. 31300716 PMC6625982

[19] Delcuve GP, Khan DH, Davie JR. 2012. Roles of histone deacetylases in epigenetic regulation: emerging paradigms from studies with inhibitors. Clin Epigenetics 4:5. doi:10.1186/1868-7083-4-5. 22414492 PMC3320549

[20] Silverstein RA, Ekwall K. 2005. Sin3: a flexible regulator of global gene expression and genome stability. Curr Genet 47:1–17. doi:10.1007/s00294-004-0541-5. 15565322

[21] Grignani F, De Matteis S, Nervi C, Tomassoni L, Gelmetti V, Cioce M, Fanelli M, Ruthardt M, Ferrara FF, Zamir I, Seiser C, Grignani F, Lazar MA, Minucci S, Pelicci PG. 1998. Fusion proteins of the retinoic acid receptor-α recruit histone deacetylase in promyelocytic leukaemia. Nature 391:815–818. doi:10.1038/35901. 9486655

[22] Pflum MKH, Tong JK, Lane WS, Schreiber SL. 2001. Histone deacetylase 1 phosphorylation promotes enzymatic activity and complex formation. J Biol Chem 276:47733–47741. doi:10.1074/jbc.M105590200. 11602581

[23] Benita Y, Cao Z, Giallourakis C, Li C, Gardet A, Xavier RJ. 2010. Gene enrichment profiles reveal T-cell development, differentiation, and lineage-specific transcription factors including ZBTB25 as a novel NF-AT repressor. Blood 115:5376–5384. doi:10.1182/blood-2010-01-263855. 20410506 PMC2902135

[24] Lee S-U, Maeda T. 2012. POK/ZBTB proteins: an emerging family of proteins that regulate lymphoid development and function. Immunol Rev 247:107–119. doi:10.1111/j.1600-065X.2012.01116.x. 22500835 PMC3334328

[25] Xuan C, Wang Q, Han X, Duan Y, Li L, Shi L, Wang Y, Shan L, Yao Z, Shang Y. 2013. RBB, a novel transcription repressor, represses the transcription of HDM2 oncogene. Oncogene 32:3711–3721. doi:10.1038/onc.2012.386. 22926524

[26] Mao R, Nie H, Cai D, Zhang J, Liu H, Yan R, Cuconati A, Block TM, Guo J-T, Guo H. 2013. Inhibition of hepatitis B virus replication by the host zinc finger antiviral protein. PLoS Pathog 9:e1003494. doi:10.1371/journal.ppat.1003494. 23853601 PMC3708887

[27] He Q, Li W, Ren J, Huang Y, Huang Y, Hu Q, Chen J, Chen W. 2016. ZEB2 inhibits HBV transcription and replication by targeting its core promoter. Oncotarget 7:16003–16011. doi:10.18632/oncotarget.7435. 26895378 PMC4941293

[28] Chen S-C, Jeng K-S, Lai MMC. 2017. Zinc finger-containing cellular transcription corepressor ZBTB25 promotes influenza virus RNA transcription and is a target for zinc ejector drugs. J Virol 91:e00842-17. doi:10.1128/JVI.00842-17. 28768860 PMC5625503

[29] Krishna SS, Majumdar I, Grishin NV. 2003. Structural classification of zinc fingers: survey and summary. Nucleic Acids Res 31:532–550. doi:10.1093/nar/gkg161. 12527760 PMC140525

[30] Kelly KF, Daniel JM. 2006. POZ for effect – POZ-ZF transcription factors in cancer and development. Trends Cell Biol 16:578–587. doi:10.1016/j.tcb.2006.09.003. 16996269

[31] Costoya JA. 2007. Functional analysis of the role of POK transcriptional repressors. Brief Funct Genomic Proteomic 6:8–18. doi:10.1093/bfgp/elm002. 17384421

[32] Kim M-Y, Koh D-I, Choi W-I, Jeon B-N, Jeong D, Kim K-S, Kim K, Kim S-H, Hur M-W. 2015. ZBTB2 increases PDK4 expression by transcriptional repression of RelA/p65. Nucleic Acids Res 43:1609–1625. doi:10.1093/nar/gkv026. 25609694 PMC4330387

[33] Wei S, Zhang M, Zheng Y, Yan P. 2018. ZBTB16 overexpression enhances white adipogenesis and induces brown-like adipocyte formation of bovine white intramuscular preadipocytes. Cell Physiol Biochem 48:2528–2538. doi:10.1159/000492697. 30121655

[34] Rossio JL, Esser MT, Suryanarayana K, Schneider DK, Bess JW, Vasquez GM, Wiltrout TA, Chertova E, Grimes MK, Sattentau Q, Arthur LO, Henderson LE, Lifson JD. 1998. Inactivation of human immunodeficiency virus type 1 infectivity with preservation of conformational and functional integrity of virion surface proteins. J Virol 72:7992–8001. doi:10.1128/JVI.72.10.7992-8001.1998. 9733838 PMC110135

[35] Lee Y-M, Wang Y-T, Duh Y, Yuan HS, Lim C. 2013. Identification of labile Zn sites in drug-target proteins. J Am Chem Soc 135:14028–14031. doi:10.1021/ja406300c. 24010488

[36] Arthur LO, Bess JWJ, Chertova EN, Rossio JL, Esser MT, Benveniste RE, Henderson LE, Lifson JD. 1998. Chemical inactivation of retroviral infectivity by targeting nucleocapsid protein zinc fingers: a candidate SIV vaccine. AIDS Res Hum Retroviruses 14(Suppl 3):S311–S319. 9814959

[37] Sargsyan K, Lin C-C, Chen T, Grauffel C, Chen Y-P, Yang W-Z, Yuan HS, Lim C. 2020. Multi-targeting of functional cysteines in multiple conserved SARS-CoV-2 domains by clinically safe Zn-ejectors. Chem Sci 11:9904–9909. doi:10.1039/D0SC02646H. 34094251 PMC8162115

[38] Zhou H, Cai Y, Liu D, Li M, Sha Y, Zhang W, Wang K, Gong J, Tang N, Huang A, Xia J. 2018. Pharmacological or transcriptional inhibition of both HDAC1 and 2 leads to cell cycle blockage and apoptosis via p21 Waf1/Cip1 and p19 INK4d upregulation in hepatocellular carcinoma. Cell Prolif 51:e12447. doi:10.1111/cpr.12447. 29484736 PMC6528930

[39] Beckers T, Burkhardt C, Wieland H, Gimmnich P, Ciossek T, Maier T, Sanders K. 2007. Distinct pharmacological properties of second generation HDAC inhibitors with the benzamide or hydroxamate head group. Int J Cancer 121:1138–1148. doi:10.1002/ijc.22751. 17455259

[40] Rekha RS, Rao Muvva SJ, Wan M, Raqib R, Bergman P, Brighenti S, Gudmundsson GH, Agerberth B. 2015. Phenylbutyrate induces LL-37-dependent autophagy and intracellular killing of Mycobacterium tuberculosis in human macrophages. Autophagy 11:1688–1699. doi:10.1080/15548627.2015.1075110. 26218841 PMC4590658

[41] Yang R, Yang E, Shen L, Modlin RL, Shen H, Chen ZW. 2018. IL-12+IL-18 cosignaling in human macrophages and lung epithelial cells activates cathelicidin and autophagy, inhibiting intracellular mycobacterial growth. J Immunol 200:2405–2417. doi:10.4049/jimmunol.1701073. 29453279 PMC5860987

[42] Shin D-M, Jeon B-Y, Lee H-M, Jin HS, Yuk J-M, Song C-H, Lee S-H, Lee Z-W, Cho S-N, Kim J-M, Friedman RL, Jo E-K. 2010. Mycobacterium tuberculosis Eis regulates autophagy, inflammation, and cell death through redox-dependent signaling. PLoS Pathog 6:e1001230. doi:10.1371/journal.ppat.1001230. 21187903 PMC3002989

[43] Kim YS, Silwal P, Kim SY, Yoshimori T, Jo E-K. 2019. Autophagy-activating strategies to promote innate defense against mycobacteria. Exp Mol Med 51:1–10. doi:10.1038/s12276-019-0290-7. PMC6906292 31827065

[44] Gutierrez MG, Master SS, Singh SB, Taylor GA, Colombo MI, Deretic V. 2004. Autophagy is a defense mechanism inhibiting BCG and Mycobacterium tuberculosis survival in infected macrophages. Cell 119:753–766. doi:10.1016/j.cell.2004.11.038. 15607973

[45] Xu Y, Jagannath C, Liu X-D, Sharafkhaneh A, Kolodziejska KE, Eissa NT. 2007. Toll-like receptor 4 is a sensor for autophagy associated with innate immunity. Immunity 27:135–144. doi:10.1016/j.immuni.2007.05.022. 17658277 PMC2680670

[46] Lam KKY, Zheng X, Forestieri R, Balgi AD, Nodwell M, Vollett S, Anderson HJ, Andersen RJ, Av-Gay Y, Roberge M. 2012. Nitazoxanide stimulates autophagy and inhibits mTORC1 signaling and intracellular proliferation of Mycobacterium tuberculosis. PLoS Pathog 8:e1002691. doi:10.1371/journal.ppat.1002691. 22589723 PMC3349752

[47] Kim J-J, Lee H-M, Shin D-M, Kim W, Yuk J-M, Jin HS, Lee S-H, Cha G-H, Kim J-M, Lee Z-W, Shin SJ, Yoo H, Park YK, Park JB, Chung J, Yoshimori T, Jo E-K. 2012. Host cell autophagy activated by antibiotics is required for their effective antimycobacterial drug action. Cell Host Microbe 11:457–468. doi:10.1016/j.chom.2012.03.008. 22607799

[48] Juárez E, Carranza C, Sánchez G, González M, Chávez J, Sarabia C, Torres M, Sada E. 2016. Loperamide restricts intracellular growth of Mycobacterium tuberculosis in lung macrophages. Am J Respir Cell Mol Biol 55:837–847. doi:10.1165/rcmb.2015-0383OC. 27468130

[49] Parihar SP, Guler R, Khutlang R, Lang DM, Hurdayal R, Mhlanga MM, Suzuki H, Marais AD, Brombacher F. 2014. Statin therapy reduces the Mycobacterium tuberculosis burden in human macrophages and in mice by enhancing autophagy and phagosome maturation. J Infect Dis 209:754–763. doi:10.1093/infdis/jit550. 24133190

[50] Jung J-Y, Robinson CM. 2014. IL-12 and IL-27 regulate the phagolysosomal pathway in mycobacteria-infected human macrophages. Cell Commun Signal 12:16. doi:10.1186/1478-811X-12-16. 24618498 PMC4007735

[51] Lin Y, Kuang W, Wu B, Xie C, Liu C, Tu Z. 2017. IL-12 induces autophagy in human breast cancer cells through AMPK and the PI3K/Akt pathway. Mol Med Rep 16:4113–4118. doi:10.3892/mmr.2017.7114. 28765958

[52] Bacon CM, McVicar DW, Ortaldo JR, Rees RC, O'Shea JJ, Johnston JA. 1995. Interleukin 12 (IL-12) induces tyrosine phosphorylation of JAK2 and TYK2: differential use of Janus family tyrosine kinases by IL-2 and IL-12. J Exp Med 181:399–404. doi:10.1084/jem.181.1.399. 7528775 PMC2191828

[53] Bromberg JF. 2001. Activation of STAT proteins and growth control. BioEssays 23:161–169. doi:10.1002/1521-1878(200102)23:2<161::AID-BIES1023>3.0.CO;2-0. 11169589

[54] Bacon CM, Petricoin EF, Ortaldo JR, Rees RC, Larner AC, Johnston JA, O'Shea JJ. 1995. Interleukin 12 induces tyrosine phosphorylation and activation of STAT4 in human lymphocytes. Proc Natl Acad Sci U S A 92:7307–7311. doi:10.1073/pnas.92.16.7307. 7638186 PMC41328

[55] Visekruna A, Volkov A, Steinhoff U. 2012. A key role for NF-κB transcription factor c-Rel in T-lymphocyte-differentiation and effector functions. Clin Dev Immunol 2012:239368–239369. doi:10.1155/2012/239368. 22481964 PMC3310234

[56] Ouyang Q, Zhang K, Lin D, Feng CG, Cai Y, Chen X. 2020. Bazedoxifene suppresses intracellular Mycobacterium tuberculosis growth by enhancing autophagy. mSphere 5:e00124-20. doi:10.1128/mSphere.00124-20. 32269154 PMC7142296

[57] Kolloli A, Subbian S. 2017. Host-directed therapeutic strategies for tuberculosis. Front Med 4:171. doi:10.3389/fmed.2017.00171. PMC5651239 29094039

[58] Jayaswal S, Kamal MA, Dua R, Gupta S, Majumdar T, Das G, Kumar D, Rao KVS. 2010. Identification of host-dependent survival factors for intracellular Mycobacterium tuberculosis through an siRNA screen. PLoS Pathog 6:e1000839. doi:10.1371/journal.ppat.1000839. 20419122 PMC2855445

[59] Lachmandas E, Beigier-Bompadre M, Cheng S-C, Kumar V, van Laarhoven A, Wang X, Ammerdorffer A, Boutens L, de Jong D, Kanneganti T-D, Gresnigt MS, Ottenhoff THM, Joosten LAB, Stienstra R, Wijmenga C, Kaufmann SHE, van Crevel R, Netea MG. 2016. Rewiring cellular metabolism via the AKT/mTOR pathway contributes to host defence against Mycobacterium tuberculosis in human and murine cells. Eur J Immunol 46:2574–2586. doi:10.1002/eji.201546259. 27624090 PMC5129526

[60] Bai X, Feldman NE, Chmura K, Ovrutsky AR, Su W-L, Griffin L, Pyeon D, McGibney MT, Strand MJ, Numata M, Murakami S, Gaido L, Honda JR, Kinney WH, Oberley-Deegan RE, Voelker DR, Ordway DJ, Chan ED. 2013. Inhibition of nuclear factor-kappa B activation decreases survival of Mycobacterium tuberculosis in human macrophages. PLoS One 8:e61925. doi:10.1371/journal.pone.0061925. 23634218 PMC3636238

[61] Wang X, Tang X, Zhou Z, Huang Q. 2018. Histone deacetylase 6 inhibitor enhances resistance to Mycobacterium tuberculosis infection through innate and adaptive immunity in mice. Pathog Dis 76. doi:10.1093/femspd/fty064. 30085165

[62] Moreira JD, Koch BV, van Veen S, Walburg KV, Vrieling F, Mara Pinto Dabés Guimarães T, Meijer AH, Spaink HP, Ottenhoff THM, Haks MC, Heemskerk MT. 2020. Functional inhibition of host histone deacetylases (HDACs) enhances in vitro and in vivo anti-mycobacterial activity in human macrophages and in zebrafish. Front Immunol 11:36. doi:10.3389/fimmu.2020.00036. 32117228 PMC7008710

[63] Rao M, Valentini D, Zumla A, Maeurer M. 2018. Evaluation of the efficacy of valproic acid and suberoylanilide hydroxamic acid (vorinostat) in enhancing the effects of first-line tuberculosis drugs against intracellular Mycobacterium tuberculosis. Int J Infect Dis 69:78–84. doi:10.1016/j.ijid.2018.02.021. 29501835

[64] Martin A, Camacho M, Portaels F, Palomino JC. 2003. Resazurin microtiter assay plate testing of Mycobacterium tuberculosis susceptibilities to second-line drugs: rapid, simple, and inexpensive method. Antimicrob Agents Chemother 47:3616–3619. doi:10.1128/aac.47.11.3616-3619.2003. 14576129 PMC253784

[65] Mosmann T. 1983. Rapid colorimetric assay for cellular growth and survival: application to proliferation and cytotoxicity assays. J Immunol Methods 65:55–63. doi:10.1016/0022-1759(83)90303-4. 6606682

[66] Morris GM, Huey R, Lindstrom W, Sanner MF, Belew RK, Goodsell DS, Olson AJ. 2009. AutoDock4 and AutoDockTools4: automated docking with selective receptor flexibility. J Comput Chem 30:2785–2791. doi:10.1002/jcc.21256. 19399780 PMC2760638

